# Diversity of *Sinorhizobium (Ensifer) meliloti* Bacteriophages in the Rhizosphere of *Medicago marina*: Myoviruses, Filamentous and N4-Like Podovirus

**DOI:** 10.3389/fmicb.2020.00022

**Published:** 2020-01-24

**Authors:** María Teresa Cubo, Cynthia Alías-Villegas, Eduardo Balsanelli, Dany Mesa, Emanuel de Souza, María Rosario Espuny

**Affiliations:** ^1^Departamento de Microbiología, Facultad de Biología, Universidad de Sevilla, Seville, Spain; ^2^Department of Biochemistry and Molecular Biology, Universidade Federal do Paraná, Curitiba, Brazil

**Keywords:** bacteriophages, P_ort11, *Podoviridae*, N4-like phage, *Sinorhizobium/Ensifer meliloti*, filamentous phage, *Medicago marina*

## Abstract

Using different *Sinorhizobium meliloti* strains as hosts, we isolated eight new virulent phages from the rhizosphere of the coastal legume *Medicago marina*. Half of the isolated phages showed a very narrow host range while the other half exhibited a wider host range within the strains tested. Electron microscopy studies showed that phages M_ort18, M_sf1.2, and M_sf3.33 belonged to the *Myoviridae* family with feature long, contractile tails and icosaedral head. Phages I_sf3.21 and I_sf3.10T appeared to have filamentous shape and produced turbid plaques, which is a characteristic of phages from the *Inoviridae* family. Phage P_ort11 is a member of the *Podoviridae*, with an icosahedral head and a short tail and was selected for further characterization and genome sequencing. P_ort11 contained linear, double-stranded DNA with a length of 75239 bp and 103 putative open reading frames. BLASTP analysis revealed strong similarities to *Escherichia* phage N4 and other N4-like phages. This is the first report of filamentous and N4-like phages that infect *S. meliloti.*

## Introduction

*Sinorhizobium* (*Ensifer*) *meliloti* is a soil bacterium able to induce the formation of root nodules on *Medicago*, *Melilotus* and *Trigonella* legumes, where these bacteria fix atmospheric nitrogen (Béna et al., 2005; Willems, 2006; Gibson et al., 2008; Zhang et al., 2012). The symbiotic relationship established between bacteria and legumes is important for sustainable agriculture because the conversion of dinitrogen to ammonia improves the overall productivity of crops without the need of adding nitrogenous fertilizers (Pérez-Montaño et al., 2014). In addition, some recent studies have shown the use of *S. meliloti-Medicago* for bioremediation purposes on soils contaminated with heavy metals (Fan et al., 2011; Alías-Villegas et al., 2015).

The most abundant and genetically diverse entities on Earth are viruses of bacteria (bacteriophages), with the global count estimated to be greater than 10^31^ (Hendrix, 2002; Clokie et al., 2011). It is well-known that bacterial species can be infected by several different phages, which can serve as agents of gene mobilization across bacterial population (Canchaya et al., 2003). Moreover, the reports of the genome sequence from a large number of bacteria have shown that many of them harbor prophages as well as genes from phages (Casjens, 2003; Decewicz et al., 2017). In the decade of the 1970s and 1980s of the 20th century some papers about morphological description of several *S. meliloti* phages (sinorhizobiophages) were published (Krsmanovic-Simic and Werquin, 1973; Werquin et al., 1988). Initially, interest in sinorhizobiophages was based on their use as molecular genetic tools due to their transducing ability, as well as their use for phage typing of indigenous rhizobia (Kowalski, 1967; Casadesús and Olivares, 1979; Sik et al., 1980; Finan et al., 1984; Bromfield et al., 1986; Kankila and Lindström, 1994). New genome sequences and structural analysis have provided a broader and deeper knowledge about the diversity of *S. meliloti* phages (Ganyu et al., 2005; Schulmeister et al., 2009; Deak et al., 2010; Brewer et al., 2014; Dziewit et al., 2014; Stroupe et al., 2014; Crockett et al., 2015; Hodson et al., 2015; Johnson et al., 2015, 2017; Schouten et al., 2015; Decewicz et al., 2017).

It is clear that phages play major roles in the ecological balance of microbial life. In the context of the symbiotic N_2_-fixation bacteria, phages that infect rhizobia may have a great impact on the population dynamics of these bacteria in the rhizosphere environment by altering, for instance, the relative numbers of resistant and/or susceptible rhizobial strains in soil (Mendum et al., 2001; Sharma et al., 2002; Malek et al., 2009). Preliminary studies on *Medicago marina* from two polluted areas of south-western Spain allowed the isolation and characterization of several new *S. meliloti* strains that showed tolerance to different stresses including salinity, alkalinity and heavy metals (Alías-Villegas et al., 2015). In order to obtain a full understanding of the biology of those sinorhizobia strains and their relationship to the plant, we studied phages from the rhizosphere sand-soil of *M. marina*. In this work we identified eight novel bacteriophages using different *S. meliloti* strains as trapping host and the rhizosphere harbors a broad diversity of phages not yet shown before.

## Materials and Methods

### Bacterial Strains, Media, and Growth Conditions

The bacterial strains used in this work are listed in Table 1. Most of the *S. meliloti* strains were isolated from nodules of *M. marina* in the south-west region of Spain, as previously reported (Alías-Villegas et al., 2015). All bacterial strains were routinely grown at 28°C on tryptone-yeast (TY) medium (Beringer, 1974). When appropriate, gentamicin was used at a final concentration of 10 μg ml^–1^.

**TABLE 1:** Bacteria and *S. meliloti* phages used in this study.

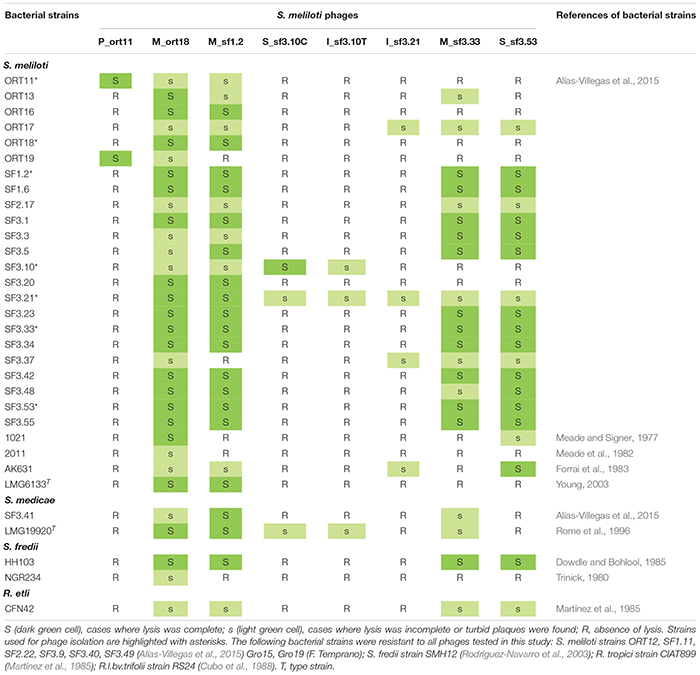

### Bacteriophage Isolation, Purification, and Propagation

Bacteriophages were isolated from *M. marina-*rhizosphere sand samples from Odiel river marshes (Huelva, Spain, 37° 10′ 28.5′′ N, 6° 55′ 51.5′′ W) using a multiple-enrichment protocol (Barnet, 1972) with some modifications. Soil samples (5 g) were mixed with 10 ml of TY and incubated at 28°C for 24 h. Soil particles were removed by centrifugation (3,000 *g*, 15 min, two times) and finally the samples were filter sterilized. Two different sets of 8 overnight *S. meliloti* strains cultures (100 μl of each bacteria in 1 ml of TY) were mixed with 4 ml of soil-sample and incubated at 28°C (180 rpm) for 3 days. Bacterial cells were then removed by centrifugation, and the supernatant was filtered through 0.45 μm Millipore filter. Four ml of this solution was used to inoculate a fresh set of bacteria as above indicated, finally yielding a solution presumably containing phages. The presence of phages was tested using the double agar overlay plaque assay (Kropinski et al., 2009). Briefly, 100 μl of phage solution were added to 200 μl of middle log-phage *S. meliloti* strains, the mixture was incubated 5 min at room temperature and then mixed with soft agar to form a lawn on TY solid medium. Plates were incubated at 28°C until plaques became visible. Single plaques were picked with a sterile toothpick, and replated three times in order to ensure the purity of the phage stocks.

High-titer phage lysates were obtained by infecting 5-ml cultures of *S. meliloti* strains in TY medium at an optical density at 600 nm (OD_600_) of 0.3 with 500 μl of phage solution and incubating at 28°C (180 rpm) for 24–48 h. Cell debris were removed by centrifugation, and the resulting supernatants were filtered twice through a 0.45 μm Millipore filter. Phage stocks were stored at 4°C.

### Electron Microscopy

For transmission electron microscopy, aliquots of high-titer lysates (20 μl) were absorbed onto 300-mesh copper grids with collodion support films for 3 min and left to dry for 24 h. Then the samples were negatively stained with 2% (w/v) uranyl acetate for 10 min. Phages were examined by transmission electron microscopy in Centro de Investigación, Tecnología e Innovación (CITIUS, University of Seville) using a Zeiss Libra 120 TEM at 80 kV.

### Host Range Determination

Host range of the isolated phages was tested as described previously (Kankila and Lindström, 1994), with some modifications. Briefly, a total of 43 rhizobial strains were tested for infection with the eight phages isolated in this work (Table 1). Top agar overlays containing 3.5 ml of 0.4% (wt/v) TY and 200 μl of an overnight culture of the bacterial strain to be tested were poured onto a solid TY plate and allowed to set. The lysates used had a phage titer of *ca* 1 × 10^9^ pfu (plaque forming units) ml^–1^ except the lysates from phages S_sf3.10C and I_sf3.10T, which reached titers of *ca* 1 × 10^5^ pfu and 1 × 10^7^ pfu ml^–1^, respectively. A 10 μl sample of three serial dilutions, from undiluted to 100-times-diluted, of the lysate of phages was spotted onto each bacterial overlay, and the plates were incubated 2 days at 28°C. After incubation, each plate was scored for lysis. Assays were performed in triplicate.

### P_ort11-Phage Adsorption

Phage adsorption experiments were carried out as described previously (Hyman and Abedon, 2009), with some modifications. Briefly, overnight bacterial cultures were adjusted to an OD_600_ of 0.05 in 5 ml TY and grown in an orbital shaker (180 rpm) at 28°C. At an OD_600_ of 0.1 phages were added at a multiplicity of infection (MOI) of 0.01, mixed briefly and placed on a rocker platform shaker at 28°C. Samples (100 μl) were removed 5, 10, 15, 20, and 25 min and added to 900 μl of phage buffer and 30 μl of chloroform, mixed for 10 s and centrifuged at 13,000 *g* for 1 min. The supernatant was removed and titrated as described above to determine the number of unadsorbed phage particles. Adsorption of phage to bacteria was measured by the number of pfu ml^–1^ remaining in the supernatant. Adsorption rates are presented as adsorption constants (*k*) and are specific for a given phage, host, and physical and chemical adsorption conditions. *k* = 2.3/B*t*⋅logP_0_/P (ml/min), being B the concentration of bacterial phages, and *t* the time interval in which the titer falls P_0_ (original) to P (final) (Hyman and Abedon, 2009).

### Bacterial Growth Curve and P_Ort11-Phage Infection

The capacity of phage P_ort11 to lyse host bacteria in liquid cultures was carried out as described by Petty et al. (2006). Briefly, bacterial growth was determined by measuring OD_600_ using a spectrophotometer. Overnight cultures of *S. meliloti* ORT11 were diluted to OD_600_ 0.05 in 3 ml prewarmed TY in 10 ml glass tubes and incubated in an orbital shaker (180 rpm) at 28°C. The OD_600_ was measured each 30 min. Phages were added to tubes to get MOIs of 1.0 or 0.1 when the cells were in early exponential phase (OD_600_ 0.1).

### One-Step Growth Curve and Burst Size

Burst size and latent period of P_ort11 were determined by the one-step growth curve as previously described (Kropinski, 2018). Briefly, an overnight bacterial culture was adjusted to an OD_600_ of 0.05 in 25 ml TY and grown in an orbital shaker (180 rpm) at 28°C. At an OD_600_ of 0.1, 10 ml of the bacterial culture were transferred to a new glass tube and phage samples were added at a MOI of 0.01. The same amount of phages was also added to 10 ml of TY as a negative control. Phages were allowed to adsorb for 5 min at room temperature, and 100 μl were transferred to a second glass tube containing 9.9 ml of TY (Tube A, 10^–2^ sample dilution), and this was repeated once more (Tube B, 10^–4^ sample dilution). Every 10 min, samples were taken from both tubes, and centrifuged at 13,000 *g* for 1 min. The supernatant was removed and titrated to determinate the number of pfu. The final growth curve represents the number of phages per initial infectious center.

### Sensitivity of P_Ort11-Phage Particles to Temperature, pH and NaCl Concentration

The sensitivity of phages to temperature, pH, and salinity was determined as described by Malek et al. (2009), with some modifications. Briefly, phages from a high titer stock were diluted 10 times in SM buffer (Kropinski et al., 2009) at pH 4.5, 7.5 or 8.5 and incubated at 4°C, 28°C or 37°C for 7 days. For the sensitivity to NaCl, phages were also diluted 10 times in SM containing NaCl at concentrations of 300, 600 or 800 mM and incubated as above for 7 days. The titer of phages pre- and post-exposure was determined as previously mentioned. Each assay was performed in triplicate and the values represented are the means.

### Phage DNA Isolation and Restriction Analysis

Bacteriophage DNA was isolated from high-titer lysates obtained from liquid infection following the PEG 8000 precipitation (Sambrook and Russell, 2001) or using the Phage DNA Isolation Kit (Norgen Biotek). Once isolated, the DNA was submitted to digestion with a range of restriction enzymes, and the results were monitored by electrophoresis on 0.8% agarose gels.

### Genome Sequencing and Bioinformatics Analysis

Sequencing libraries were constructed using Nextera XT kit (Illumina), according to the manufacturer,s recommendations. The libraries were quantified, and quality verified with Bioanalyzer High Sensitivity DNA Kit (Agilent). The libraries were diluted to 500 pM and pooled. This pool was quantified by qPCR using the Kapa Biosystems kit, and 17.5 pM of pooled libraries were sequenced in the Illumina MiSeq with MiSeq Reagent 500V2 kit, generating paired reads of 250 bases. The sequence data set were *de novo* assembled using CLC Workbench 8.0 (Qiagen). Sequence annotation was performed using the programs PhAnToMe^1^, RAST^2^, Phaster (Arndt et al., 2016), and BLASTx^3^ (Altschul et al., 1990). Translated ORF sequences were compared with known proteins using standard protein-protein BLASTP (see text Footnote 3) (Altschul et al., 1990). Genes encoding the putative tRNAs were analyzed using tRNAScan-SE (Lowe and Chan, 2016).

### Phylogenetic Analyses

The following protein sequences (accession numbers in parentheses) were downloaded from the GenBank data-base^4^ : *Escherichia* virus N4 (EF056009.1), *Escherichia* phage vB_EcoP_G7C (NC_015933.1), *Escherichia* phage phi G17 (MH358458.1), *Escherichia* phage vB_EcoP_PhAPEC7 (NC_024790.1), *Escherichia* phage vB_EcoP_PhAPEC5 (NC_024786.1), *Escherichia* phage PGN829.1 (MH733496.1), *Escherichia* phage IME11 (NC_019423), *Escherichia* phage St11Ph5 (MG208881.1), *Escherichia* phage EC1-UPM (KC206276.2) *Escherichia* phage ECBP1 (NC_018854), *Escherichia* phage OLB145 (MH992123.1), *Escherichia* phage PMBT57 (MG770228.1), *Escherichia* phage Bp4 (NC_024142), *Achromobacter* phage phiAxp-3 (NC_028908.2), *Achromobacter* phage JWAlpha (NC_023556), *Achromobacter* phage JWDelta (KF787094.1), *Erwinia* phage Ea9-2 (NC_023579), *Erwinia* phage phiEaP-8 (MH160392.1), *Erwinia* phage vB_EamP-S6 (NC_019514), *Erwinia* phage vB_EamP_Frozen (NC_031062.2), *Erwinia* phage vB_EamP_Rexella (KX098390.2), *Klebsiella* phage KP8 (MG922974.1), *Pseudomonas* phage inbricus (MG018928.1), *Shigella* phage pSb-1 (NC_023589.1), *Xanthomonas* phage RiverRider (MG983743.2). The phylogenetic analyses were performed using the program CLUSTALW in the MEGA5 software package (Tamura et al., 2011) with the neighbor-joining algorithm (Saitou and Nei, 1987) method. Tree robustness was assessed by bootstrap resampling (1,000 replicates each).

## Results and Discussion

### Isolation of Bacteriophages

Following the enrichment and isolation procedure (Barnet, 1972) with seven strains of *S. meliloti*, eight lytic phages were isolated from the rhizosphere sand-soil of *M. marina*. The phages were named using the nomenclature suggested by Aziz et al. (2018) as follows: vB_SmeP_ort11 and vB_SmeM_ort18 (ort, from Odiel and Riotinto, where the host bacterial strains were isolated), which infected *S. meliloti* strains ORT11 and ORT18 respectively; vB_SmeM_sf1.2, vB_SmeI_sf3.21, vB_SmeM_sf3.33, and vB_SmeS_sf3.53 (sf, San Fernando) which infected *S. meliloti* strains SF1.2, SF3.21, SF3.33, and SF3.53, respectively; vB_SmeS_sf3.10C, and vB_SmeI_sf3.10T, both of which infected the strain SF3.10 of *S. meliloti.*

The plaques formed by all phages, except P_ort11, in the lawns of the host strains were as small as a pinhead when a 0.7% lawn overlay was used. For this reason, decreasing concentrations of TY-agar (%, w/v) in the top lawn overlays were tested for each phage and the 0.3% was routinely used obtaining the plaque sizes of 0.5–2.6 mm diameter. For phage P_ort11 0.4% TY-agar was used routinely and the plaque sizes varied from 1.9–2.5 mm of diameter. The appearance of the plaques also varied: P_ort11, M_ort18, M_sf1.2, S_sf3.10C, M_sf3.33, and S_sf3.53 formed clear plaques and I_sf3.10T, and I_sf3.21 formed turbid plaques (Supplementary Figure S1).

### Host Range

Bacteriophages are frequently used for identification and grouping of related bacterial strains due the narrow host range they usually show (Malek et al., 2009; Santamaría et al., 2014; Amgarten et al., 2017). Thirty-five *Sinorhizobium meliloti* strains, two *S. medicae*, including type strains, 3 *Sinorhizobium fredii* strains, and 3 *Rhizobium* species were tested for susceptibility to each of the phages isolated in this work (Table 1). A strain was judged as “highly susceptible” if the 10^–2^ dilution of the phage stock produced plaques or confluent lysis; “less susceptible” if the undiluted stock produced low density of plaques; and “resistant” if no effect was observed (Supplementary Figure S2). Based on the number of bacterial strains infected by the isolated phages, two groups could be established (Table 1). One group, formed by phages M_ort18, M_sf1.2, M_sf3.33, and S_sf3.53, showed a wide host range, infecting 74, 63, 49, and 47% of the 43 rhizobial strains tested, respectively. In contrast, the other group of phages showed a very narrow host range, P_ort11 infected only two strains, S_sf3.10C and I_sf3.10T infected three and I_sf3.21 infected four of the bacterial strains tested. Usually when the host range is studied in a group of isolated phages, some of them are able to infect several bacterial strains while others are more specific or even infect a single strain within a species (Hyman and Abedon, 2010).

From the host point of view, most of the *S. meliloti* and *S. medicae* strains were susceptible to some of the phages tested, varying from 1 to 7 phages. It is striking that strains of different species from the *S. meliloti* used as phage-host, such as *S. fredii* HH103 and NGR234, or different genus, as *Rhizobium etli* CFN42, were also susceptible to the sinorhizobiophages M_ort18, M_sf1.2, M_sf3.33, and S_sf3.53. Possibly these phages might adsorb to receptors present in the outer membrane that are frequently present in rhizobia. The LPS of Gram-negative bacteria are frequently used as receptor for many phages, for instance for the podovirus P22 (Rakhuba et al., 2010). Crook et al. (2013) have reported that some *Sinorhizobium* phages, including the podovirus ΦM5 and ΦM6, are dependent on lipopolysaccharides (LPS) and/or the porin RopA1 (rhizobial outer membrane protein A). This protein was initially reported in *Rhizobium leguminosarum* bv. *viciae* 248 (de Maagd et al., 1992) but it is also present in *S. meliloti* 1021 (*SMc02396*), *S. fredii* strains HH103 (*SFHH103_00750*) and NGR234 (*NGR_a03720*). Further work would be necessary to study this process in detail.

### Phage Morphology

**FIGURE 1:**
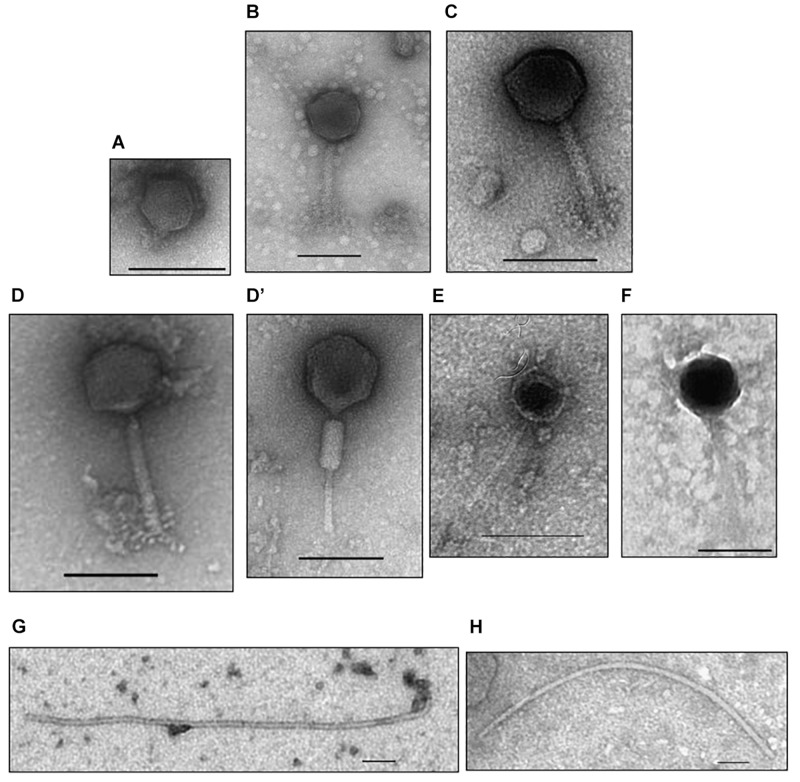
Electron micrographs of *Sinorhizobium meliloti* bacteriophages isolated from the rhizosphere of the coastal legume *Medicago marina*. **(A)** P_ort11, **(B)** M_ort18, **(C)** M_sf1.2, **(D,D’)** M_sf3.33, **(E)** S**_**sf3.10C, **(F)** S_sf3.53, **(G)** I_sf3.10T, **(H)** I_sf3.21. Phage particles were negatively stained with uranyl acetate. Scale bars represent 100 nm.

Morphology features for the eight virions were assessed by transmission electron microscopy. As can be seen in Figure 1 three morphological types were distinguished. Phages M_ort18, M_sf1.2, and M_sf3.33 have an icosaedral head (70–86 nm in diameter) with a long contractile tail (100–125 nm long, 10–14 nm wide), characteristics typical of *Myoviridae* family (Ackermann and Prangishvili, 2012). Phages S_sf3.10C and S_sf3.53 exhibited a polyhedrical head and a long and thin tail, probably belonging to the family *Siphoviridae*, though the electron microscopy images were not sharp enough to confirm such allocation. Phage P_ort11 showed characteristic features of the family *Podoviridae*, namely an icosahedral head (54 nm diameter) with a short tail. In contrast to these head-tail structures, the third type of morphology was represented by phages I_sf3.10T and I_sf3.21, which showed a flexible filamentous shape, thus resembling members of *Inoviridae* family. To the best of our knowledge, this is the first report of filamentous phages infecting *S. meliloti* strains. Filamentous phages are relatively rare; therefore, the rate of their discovery is low (Ackermann and Prangishvili, 2012).

### Biological Characterization of P_ort11

**FIGURE 2:**
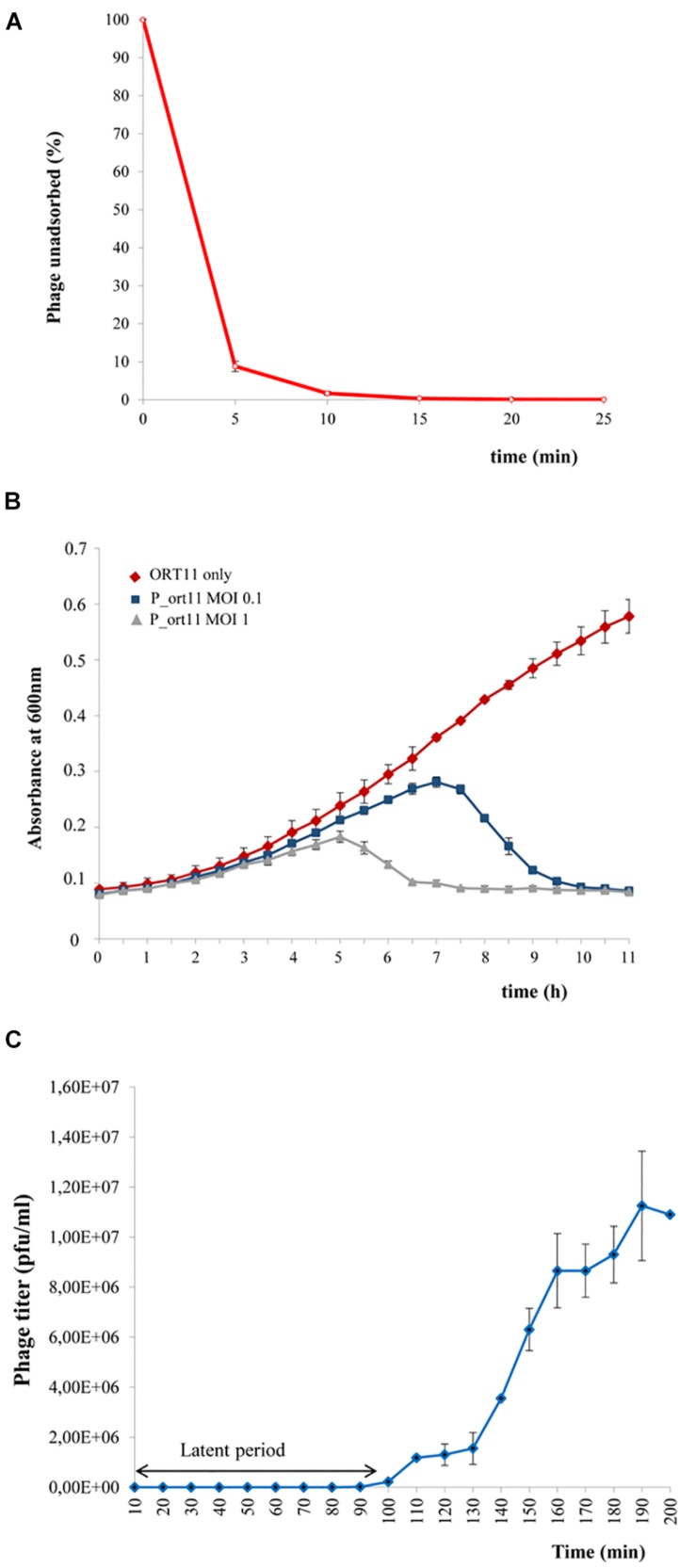
Biological characterization of P_ort11. **(A)** Adsorption of P_ort11 to ORT11 at a MOI 0.1. The supernatant was titrated at various time points to determinate the amount of phages unadsorbed. **(B)** *S. meliloti* ORT11 growth curves in the presence and the absence of P_ort11. Phage was added at MOIs of 0.1 and 1 to ORT11 in the early exponential growth phase (OD600 0.1). **(C)** One-step growth curve of P_ort11. ORT11 bacteria were infected with a MOI 0.01. Each graph shows an average of three independent experiments, including standard error.

Since phage P_ort11 showed the narrowest host range and sinorhizobiophages of the *Podoviridae* family are poorly known we decided to choose this phage for further characterization. First, we studied the capacity of phage P_ort11 to lyse host bacteria in liquid cultures by addition of phages at different MOIs to a culture of *S. meliloti* strain ORT11 in early exponential growth. Infection with phages at a MOI of 0.1 produced a slight reduction of the growth rate followed by a cessation of growth and bacterial lysis 6 h after P_ort11 addition (Figure 2A). Similar results were obtained when the phage was added at a MOI of 1, although cessation of growth and bacterial lysis occurred more rapidly, 3 h after P_ort11 addition (Figure 2A). That is, bacterial lysis happened faster when a higher concentration of phages is added. No resumption of bacterial growth was observed after a prolonged incubation period (data not shown) independently of the MOI used, whereas other bacteria have the ability to resume growth after varying times of incubation in the presence of the phage even a high MOI (Petty et al., 2006; Jun et al., 2014; Peng and Yuan, 2018).

Phage adsorption assays showed that P_ort11 adsorbed rapidly to *S. meliloti* ORT11 (Figure 2B), with 91.13% of phages adsorbed in 5 min, displaying an apparent maximum at 15 min post mixing. The adsorption constant (Kropinski, 2009) calculated for this phage was *k* = 5.44⋅10^–9^ ml min^–1^. This constant is specific for each phage and the greater the *k* the earlier the phage adsorption. *k* gives also an idea about, for instance, the abundance of receptor(s) present on a cell surface. Kropinski (2009) has pointed out that differences in *Escherichia coli* phages as T4 with a *k* of 2.4 × 10^–9^ml min^–1^ and M13 phage with a *k* 3 × 10^–11^ ml min^–1^ are due to the fact that T4 recognizes several hundred of receptor sites per cell whereas M13 binds only to the tip of F pili. Therefore, from the *k* calculated for P_ort11 it could be deduced that the number of receptors for this phage may be high in the bacterium ORT11.

The multiplication rate, which is another essential feature of the life cycle of phages, can be calculated by two parameters: the latency (the period between the adsorption and the beginning of lysis of the host bacterium) and the burst size (the number of particles released in one cycle of infection). To obtain both data one-step growth curve of phage P_ort11 was carried out. For this, *S. meliloti* ORT11 at early exponential growth was infected with the phage at a MOI of 0.01. The latent period of the phage was approximately 90 min (Figure 2C), and the average burst size was about 19–20 pfu per bacterial cell.

### Thermal, pH and Salt Stability

**FIGURE 3:**
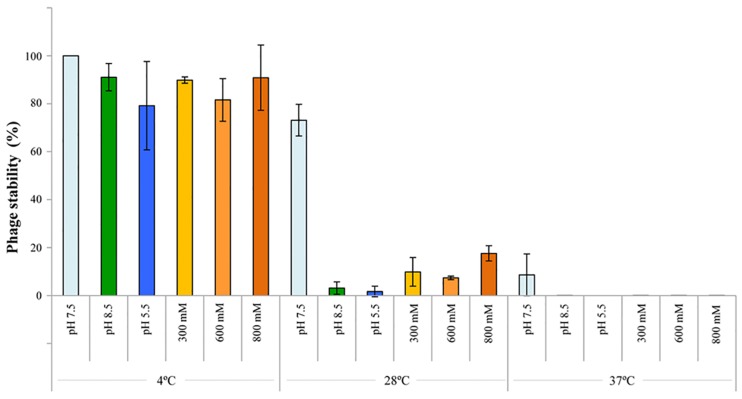
Thermal/pH/salinity sensitivities of P_ort11. To study the sensitivity, the phage was incubated at those conditions for 1 week. The titer of phages pre- and post-exposure was determined. Optimal conditions (4°C, pH 7.5, ClNa 100 mM) acted as control. The values indicate the means of results from three independent experiments, including standard error.

The stability of phage P_ort11 was assessed by calculating the pfu changes under different pH, temperature and salinity conditions. The phage showed more sensitivity to temperature than to pH or salinity (Figure 3). Thus, P_ort11 was highly stable at lower temperatures on the range of pHs values or salt concentrations studied, although there was a slight reduction in the pfu at pH5.5. The phage was extremely unstable at higher temperatures. At neutral pH and 28°C or 37°C the titer of the phage underwent a 27 and 91% reduction, respectively, while the viability of the phage decreased sharply with the combination of acidic/alkaline pHs or salinity and high temperatures, exhibiting a 0% of viability at pH 5.5 at 37°C (Figure 3 and Supplementary Table S1).

In general, phages tend to be more stable at a wide range of pHs than at different temperatures, in fact high temperatures cause the loss of phage viability (Jun et al., 2014; Kwiatek et al., 2017; Peng and Yuan, 2018), although Malek et al. (2009) reported opposite results for 3 rhizophages (ΦRP1, ΦRP2, and ΦRP3) specific for *Robinia pseudoacacia* which were stable at 37°C and very sensitive at 4°C.

### Genomic Analysis of P_ort11

The DNA of P_ort11 was completely digested by DNase I and with restriction enzymes with different methylation sensitivities (*Bsr*BI, *Bam*HI, *Bgl*I, *Hind*III, *Msp*I, *Nde*I, *Nru*I, *Xba*I) but not by RNase A. Highly modified genomes are common in rhizophages to avoid their cleavage by restriction enzymes present in the host (Finan et al., 1984; Martin and Long, 1984; Swinton et al., 1985; Mendum et al., 2001; Santamaría et al., 2014). Similar results were obtained with some phages analyzed in this study (M_ort18, M_sf1.2, M_sf3.33, data not shown), however, DNA from P_ort11 did not exhibit any noticeable resistance to restriction digestion. The genome of this phage was determined to be linear dsDNA through *Exo*III/*Nde*I double digest treatment (data not shown), with its size determined by whole-genome sequencing being 75.2 kb (accession number MN228696), comparable to all N4-like phages known so far (Wittmann et al., 2015). The GC content was 44.2 % in contrast to its host, *S. meliloti* ORT11, which has a GC content of 61.9%, a feature that also occurs in other N4-like phage genomes (Wittmann et al., 2014; Amgarten et al., 2017).

The genome is close-packed, 94.1% of the genome being occupied by coding sequences. Gene prediction using different servers (see Materials and Methods) identified 103 putative CDSs, most of which were initiated by an ATG codon (90.3%) and only 9.7% by GTG as start codon. Short overlaps between two coding regions are frequent. Five transfer RNA (tRNA) genes were identified for proline, asparagine, threonine, glutamine, and arginine (Table 2) using tRNAscan-SE (Lowe and Chan, 2016). The tRNA-anticodons present in P_ort11 were also found in the genome of the bacterial host *S. meliloti* ORT11 (bacterial sequence not published yet). The size of the putative tRNAs varied from 72 to 74 bp with a mean DNA G+C content of 51–53% except the Arg-tRNA which was 45%. The presence of tRNAs in the genomes of N4-like phages ranges from 10 in *Salmonella* phage FSL SP-058 (Moreno Switt et al., 2013) or *Pseudomonas* phage ZC03 (Amgarten et al., 2017) to no tRNA in *Achromobacter* phages JWAlpha and JWDeltha (Wittmann et al., 2014). Thereby, although phages use the bacterial translation machinery, most of N4-like phages harbor some tRNA-genes in their genomes. Possibly, some of these genes correspond to codons that either the host cell does not provide or are rare in the host genome (Bailly-Bechet et al., 2007; Wittmann et al., 2015). To know if this was the case for phage P_ort11, codon usage frequency for amino acids proline, asparagine, threonine, glutamine, and arginine was compared between phage P_ort11 and its bacterial host *S. meliloti* ORT11 (Table 2). This analysis showed that codons more frequently used by the phage for arginine, proline and glutamine were those the tRNA of which is present in its genome. The frequency of codon usage for asparagine and threonine was similar for the different possible codons. In contrast, the bacteria showed a different pattern of codon usage for these five amino acids (Table 2).

**TABLE 2:** Codon usage of *Sinorhizobium* phage P_ort11 and bacterial host *S. meliloti* ORT11 for amino acids arginine, asparagine, glutamine, proline, and threonine.

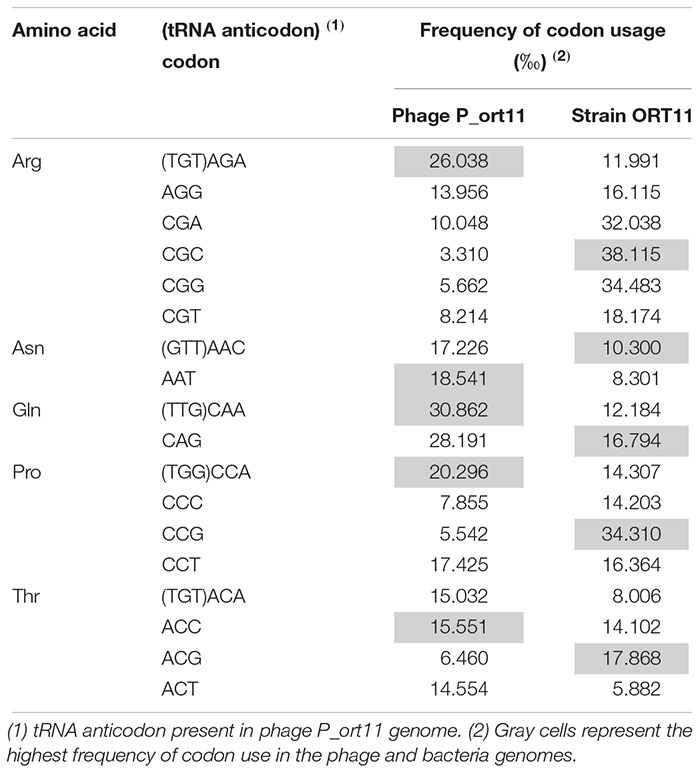

Based on sequence similarity, 42 CDSs (40.8%) shared a significant similarity to previously characterized gene products, 18 of them (17.5%) could be assigned putative functions, while 24 (23.3%) predicted protein-encoding genes showed sequence similarities to hypothetical proteins already described in other phages. Finally, 61 CDSs (59.2%) exhibited no homology to anything present in the database (Supplementary Table S2).

**FIGURE 4:**
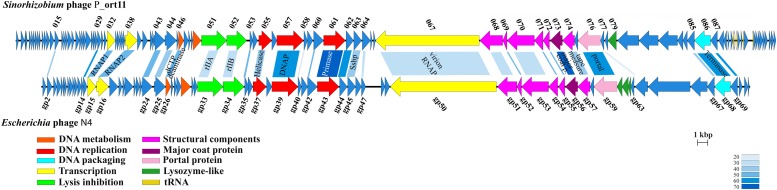
Genome map of P_ort11 and comparison with *Escherichia* phage N4. Proposal functional clusters are marked by the same color. Shading indicates the degree of amino acids sequence identity of gene products with an identity of >20%. Putative functions of genes are indicated.

The genome of P_ort11 showed significant similarity to the genome of the Podovirus *Escherichia* phage N4 (Figure 4), with 28 CDSs having amino acid identities ranging from 27 up to 68% (Table 3). The remaining CDSs showed similarity with proteins of many other N4-like phages. This is the case of N4-like *Erwinia* phages (21 CDSs), *Pseudomonas* phage inbricus (4 CDSs), *Achromobacter* phages (4 CDSs), *Klebsiella* phage Kp8 (2 CDSs), and *Enterobacter* phage Bp4 (2 CDS) (see Supplementary Table S2). Therefore, P_ort11 shows a highly mosaic genome as it has been reported for many phages, so that their genomes contain either single genes or groups of genes that are shared between individual genomes. These combinations of individual gene(s) has led the suggestion that the genome of each phage is unique (Hendrix, 2002; Pedulla et al., 2003; Morris et al., 2008; Johnson et al., 2017; Peng and Yuan, 2018).

**TABLE 3:** Comparison of *Sinorhizobium* phage P_ort11 predicted proteins and their orthologs in *Escherichia* phage N4.

**P_ort11 ORF name**	**Predicted function**	**%Identity (No. of aa/total)**	**Homologous genes in N4**
SmeP_ort11_015	transcription activation	38 (41/109)	gp2
SmeP_ort11_029	PHA00684 superfamily	60 (73/121)	gp14
SmeP_ort11_032	RNAP1	50 (133/268)	gp15
SmeP_ort11_038	RNAP2	53 (217/407)	gp16
SmeP_ort11_043	Hypothetical protein	50 (177/352)	gp24
SmeP_ort11_044	Putative metallopeptidase domain; pfam13203	42 (160/381)	gp25
SmeP_ort11_046	Putative dCTP deaminase	54 (90/166)	gp26
SmeP_ort11_051	Phage rIIA lysis inhibitor	38 (120/135)	gp33
SmeP_ort11_052	Phage rIIB lysis inhibitor	30 (160/538)	gp34
SmeP_ort11_053	Hypothetical protein	52 (50/96)	gp35
SmeP_ort11_055	ATP-dependent DNA helicase	47 (202/430)	gp37
SmeP_ort11_057	DNA polymerase I	63 (548/870)	gp39
SmeP_ort11_058	Hypothetical protein	27 (26/96)	gp40
SmeP_ort11_060	Hypothetical protein	58 (192/330)	gp42
SmeP_ort11_061	DNA primase	71 (507/713)	gp43
SmeP_ort11_062	hypothetical protein	64 (152/239)	gp44
SmeP_ort11_063	ssDNA binding protein	45 (122/270)	gp45
SmeP_ort11_064	Hypothetical protein	37 (46/123)	gp47
SmeP_ort11_067	Virion RNA polymerase	35 (1228/3553)	gp50
SmeP_ort11_068	Hypothetical protein (66 kDa protein)	32 (29/90)	gp51
SmeP_ort11_069	Hypothetical protein (16.5 kDa protein)	40 (58/144)	gp52
SmeP_ort11_070	Hypothetical protein	36 (323/905)	gp53
SmeP_ort11_071	Putative structural protein	50 (152/304)	gp54
SmeP_ort11_072	hypothetical protein	50 (99/200)	gp55
SmeP_ort11_073	Major coat protein	77 (310/401)	gp56
SmeP_ort11_074	Tape measure protein	43 (176/406)	gp57
SmeP_ort11_076	Portal protein (94 kDa protein)	64 (479/743)	gp59
SmeP_ort11_077	Conserved domain DUF460 Superfamily	27 (25/91)	gp63
SmeP_ort11_085	Hypothetical protein (30 KDa protein)	41 (94/230)	gp67
SmeP_ort11_086	Phage terminase, large subunit	68 (362/529)	gp68
SmeP_ort11_087	Hypothetical protein	63 (138/218)	gp69

#### Transcription

N4-like phages are the only known phages that harbor three genes for RNA polymerases (RNAPs) for the transcription of early and middle genes (Lenneman and Rothman-Denes, 2015). The most striking characteristic in this genus is the presence of a large virion-associated RNA polymerase (vRNA) with around 3500 amino acids inside the virion which is injected into the cell in conjunction with the phage DNA for immediate start of early gene transcription. Transcription of middle genes is initiated by two other RNA polymerases (RNAP1 and RNAP2) encoded by the phage. That is, N4 has evolved a unique transcriptional strategy that is independent from the host immediately upon infection and only the late N4 transcription is carried out by the host σ^70^-RNAP (Lenneman and Rothman-Denes, 2015). The CDS SmeP_067 of P_ort11 encoded the vRNAP of 3419 amino acids, which was 35.7% identical to that of *Klebsiella* phage KP8 and 34.6% identical to *Escherichia* virus N4. P_ort11 also carried CDSs encoding RNAP1 and RNAP2 (SmeP_032 and SmeP_038, respectively). Both genes are separated by 5 small genes as it is found in many N4-like phages (Zhao et al., 2009; Ceyssens et al., 2010; Born et al., 2011; Chan et al., 2014), whereas in phage N4 and some other N4-like phages both genes are together (Willis et al., 2002; Wittmann et al., 2014). In phage N4 the gp2 has been shown to be a protein involved in the activation of transcription by binding to single-stranded DNA at middle promoters and recruiting RNAP2. Then, N4 RNAP2 recognizes specific sequences in the template strand and initiates transcription (Carter et al., 2003; Lenneman and Rothman-Denes, 2015). The CDS SmeP_015 in P_ort11 presented 37.6% of identity to N4-gp2, which presumably might be involved in the activation of middle transcription.

#### DNA Metabolism, Replication and Packaging Genes

At least eight genes encoded by the P_ort11 genome are predicted to play roles in phage nucleotide metabolism (Table 3 and Supplementary Table S2). These include a gene encoding a deoxycytidine triphosphate deaminase with a conserved Dcd domain (COG0717) and a gene encoding a thymidylate synthase (pfam 00303). Genes involved in replication included a nucleoside triphosphate pyrophosphohydrolase (NTP-PPase superfamily, EC.3.6.1.8), a gene for an ATP-dependent DNA helicase (RecD/TraA family, cI36909), a gene encoding a DNA polymerase (DNA_polA superfamily, cI36696), a gene for a DNA primase containing a conserved domain DUF3987 (pfam13148) on the N-terminal and the PriCT_1 domain on the C-terminal (pfam08708), and a gene encoding a single-stranded DNA-binding protein (Ssb). Finally, a gene involved in DNA packaging into the capsid was predicted: a terminase large subunit protein containing a Terminase_6C domain (cI02216). Many phages use two proteins for DNA packaging forming a hetero-multimeric structure with a small subunit for DNA binding and a large subunit with an ATPase domain and an endonuclease function. However, N4-like phages have developed another strategy for DNA packaging into their capsids since they have only the large subunit (Wittmann et al., 2015). Further work would be necessary to study this process in detail.

These three clusters of genes are arranged in P_ort11 in the same order as in N4 and other N4-like phages (Figure 4). The main difference among them resides in the insertion of small genes specific for each clade of phages, suggesting that they might have evolved from a common ancestor, and later specialized to infect different group of hosts (Chan et al., 2014; Amgarten et al., 2017).

#### Mosaic Structure of Phage P_ort11 Structural Proteins and Host Lysis Gene

The genes encoding for the structural proteins formed a cluster with the same arrangement found in N4 phage. These genes are located upstream of vRNAP gene and the gene encoding for the portal protein was located at the beginning of this cluster. There were the same number of genes in the genomes both of N4 and P_ort11 (Table 3). However, each protein in P_ort11 revealed only similarities to proteins from different N4-like phages. The highest identities were observed for the major coat protein (81% from *Klebsiella* phage KP8 and 77% from N4) and the portal protein (65% from *Erwinia* phage Ea9_2 and 64% from N4). Other structural proteins showed identities with *Escherichia* phage Bp4, *Achromobacter* phage phiAxp-3 or *Erwinia* phage phiEaP-8 (Supplementary Table S2).

The gene or genes involved in cell lysis constitute a cluster less conserved among the N4-like phages. In this regard, Wittmann et al. (2014) pointed out that five different groups of clusters with apparently genus-specific strategies for host lysis could be identified. The largest group harbors genes for a holin, a N-acetylmuramidase and a Rz protein. However, P_ort11 could not be included in any of these groups. BLASTP analysis identified only one gene, SmeP_079 in P_ort11, encoding a protein that contained a lysozyme-like domain, in particular the 40% of the N-terminal of the protein was 46% identical to the lysozyme-like domain of the baseplate hub protein from *Sinorhizobium* phage N3. Since the host of this N3 phage is the same as the phage of this study, horizontal gene exchanges might have occurred from phages through the same host bacteria, which is in agreement with the mosaic model proposed by Hendrix et al. (1999).

On the other hand, in the genome of P_ort11 the deduced amino acids sequence of two genes located next to the thymidylate synthase gene showed homology to the rIIA and rIIB proteins from *Pseudomonas* phage inbricus, with low identity (28 and 34 %, respectively). Identified in phage T4, rIIA and rIIB were initially related to lysis inhibition when an *E. coli* cell was attacked by several T-even phages, although the role of those proteins is not clear. Paddison et al. (1998) suggested that in the absence of these proteins, an alternate pathway for lysis would occur. In any case, rIIA and rIIB proteins should have an important role in the life cycle of phages because most of the N4-like phages harbor genes for both proteins (Chan et al., 2014; Wittmann et al., 2015).

### Phylogenetic Analysis

**FIGURE 5:**
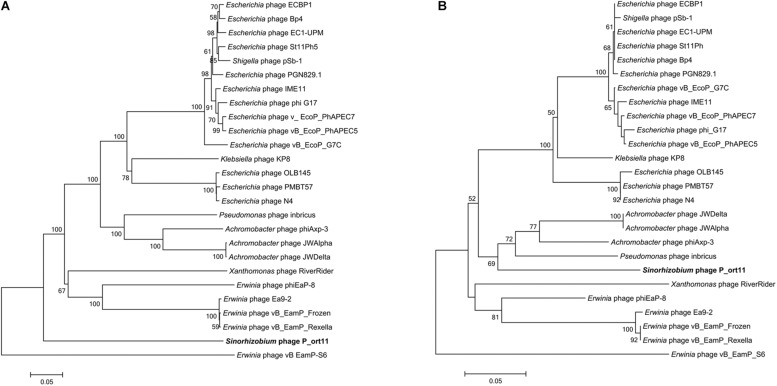
Phylogenetic analysis of vRNAP **(A)** and terminase large subunit **(B)** proteins of P_ort11 compared to other N4-like phages. Neighbor-joining tree was constructed based on ClustalW (MEGA5). Bootstrap values (>50 %) are expressed as percentages of 1,000 replicates. Bar, 0.05 amino acid substitutions per site.

Phylogenetic analysis of the *Sinorhizobium* P_ort11 and other N4-like phages based on the alignment of the most conserved structural genes products (major coat and portal proteins), DNAP, vRNAP, and terminase were carried out. Most of the trees obtained revealed that phages infecting closely related hosts were clustered together. Although P_ort11 appeared in a separate clade in most of the trees (Figure 5A and Supplementary Figure S3), it is clear that this phage belongs to the group named “N4virus” (Wittmann et al., 2015). This group of phages displays significant protein homology and members of *G7virus* (many *Escherichia* phages), *Escherichia* phage N4, *Erwinia* phage Ea9_2 and *Achromobacter* phage JWAlpha belong to the group. When the terminase large subunit protein was used for the phylogenetic analysis, the neighbor-joining tree revealed that *Sinorhizobium* P_ort11 phage was clustered together with other N4-like phages probably sharing the same mechanism for DNA packaging (Figure 5B).

### *Sinorhizobium meliloti* Virulent Bacteriophages

The presence of phages that infected rhizobia in soils where alfalfa crops were grown was demonstrated as early as 1936 (Vandecaveye and Katznelson, 1936). In 2014, it was published the first lytic *Sinorhizobium* phage ΦM12 genome sequence (Table 4), which was used in the 1980s as transducing phage for the discovery of *nod*/*fix* genes. ΦM12 is a *Myoviridae* phage belonging to a new group of T4-superfamily phages, which has features of both T4-like cyanophages and T4-like phages of enteric bacteria (Brewer et al., 2014). Since then new sinorhizobiophages genome sequences have been reported (Table 4), four of them are also *Myoviridae* phages (ΦM7, ΦM9, ΦM19, ΦN3), three *Podoviridae* (ΦM5, ΦM6, P_ort11), and for sure new will be described in the near future. The detailed study of each of these phages genome sequences is showing a genomic mosaicism combining gene clusters from different genetic lineages, but all combinations fit appropriately to infect their host, that is, *S. meliloti* strains.

**TABLE 4:** *Sinorhizobium meliloti* lytic phages whose genome has been sequenced.

**Phage**	**Size**	**% G+C**	**tRNAs**	**ORFs**	**Transducer**	**Family,**	**Host**	**Gen Bank**	**References**
	**(kbp)**					**Genus**	**(*S. meliloti***	**Accession**	
							**strain)**	**number**	
ΦM5	44.0	61	Met	65	NO	*Podoviridae* LUZ24-like	SU47	MF074189.1	Johnson et al., 2017
ΦM6	68.17	42.9	NO	122	NO	*Podoviridae* (marine phage group)	SU47	MH700630.1	Brewer et al., 2018
ΦM7	188.42	49	Thr (2), Gln, His, Met, Cys, Lys,	361	N/A	*Myoviridae*, Emdodecavirus*	SU47	KR052480.1	Schouten et al., 2015
ΦM9	149.21	50	NO	275	N/A	*Myoviridae*, T4-like Superfamily	SU47	KP881232	Johnson et al., 2015
ΦM12	194.7	49	Thr, Gln, Met, Cys, Lys	377	YES	*Myoviridae*, Emdodecavirus*	SU47	KF381361.1	Brewer et al., 2014
ΦM19	188.04	49	Thr (2), Gln, His, Met, Cys, Lys	361	N/A	*Myoviridae, Emdodecavirus*	SU47	KR052481.1	Crockett et al., 2015
ΦN3	206.71	49	Thr, Gln, His, Asn, Met,	398	YES	*Myoviridae*, Emdodecavirus*	1021	NC_028945	Hodson et al., 2015
P_ort11	75.23	44.2	Pro, Asn, Thr, Gln, Arg	103	NO	*Podoviridae*, N4-like	ORT11	MN228696	This paper

*According to updata of International Committee on Taxonomy of Viruses (ICTV) ratified in February 2019 (https://talk.ictvonline.org/taxonomy/vmr/m/vmr-file-repository/8287). N/A, not available.

The analysis of the genome-sequence showed that P_ort11 belongs to the N4-like group. Lavigne et al. (2008) reported a classification of the *Podoviridae* family of bacteriophages and they described the group “N4-like phages” as a group formed only by the *Escherichia* phage N4. Because of its unique genomic structure it was considered as a genetic orphan for many years (Choi et al., 2008; Lenneman and Rothman-Denes, 2015); however, since then, new members of the N4-like group have been identified based on the increasing number of sequenced phage genomes (Fan et al., 2012; Chan et al., 2014; Wittmann et al., 2014; Amgarten et al., 2017; Park et al., 2018).

## Conclusion

Isolation of eight new sinorhizobiophages from the rhizosphere of *M. marina* showed the wide range of bacteriophages that infect the same host, *S. meliloti*, although each phage is able to infect only certain strains of this bacterium. We established that phages M_ort18, M_sf1.2 and M_sf3.33 belong to the Order *Caudovirales*, family *Myoviridae*, with icosaedral heads and rigid, contractile tails; and phages S_sf3.10C and S_sf3.53 may belong to the Order *Caudovirales*, family *Syphoviridae*. The 6th phage (P_ort11) belongs also to the Order *Caudovirales*, but to the family *Podoviridae*, with icosaedral head and a short tail. The remaining two phages (I_sf3.10T and I_sf3.21) possibly belong to the *Inoviridae* family due to their filamentous shape. This is the first time filamentous sinorhizobiophages are described.

Analysis of the P_ort11 genome and comparisons with other genomes revealed strong similarities to the *Escherichia* phage N4 and other N4-like phages. This group of podoviruses infects many different hosts with *Sinorhizobium* as a new member. All of these phages share a highly conserved genomic structure and strong similarities at the amino acid level of core genes. Possibly, some of the differences found in these phages originated from their relationship with their host.

## Data Availability Statement

The raw data supporting the conclusion of this article will be made available by the authors, without undue reservation, to any qualified researcher.

## Author Contributions

MC designed the study, performed and analyzed the results, and wrote the manuscript. CA-V carried out the phylogenetic analyses. ME and CA-V took the sand-soil samples and suggested improvements on the manuscript. DM made the map-genome figure. EB contributed to the DNA sequencing. ES made possible the DNA sequencing and provided critical writing of the manuscript. All authors read and approved the final manuscript.

## Conflict of Interest

The authors declare that the research was conducted in the absence of any commercial or financial relationships that could be construed as a potential conflict of interest.

## Footnotes

1 http://grantome.com/grant/NSF/DBI-0850356

2 http://rast.nmpdr.org/

3 http://blast.ncbi.nlm.nih.gov

4 http://www.ncbi.nlm.nih.gov/GenBank/index.html

## References

[B1] Ackermann H.-W. Prangishvili D. (2012). Prokaryote viruses studied by electron microscopy. *Arch. Virol.* 157 1843–1849. 10.1007/s00705-012-1383-y 22752841

[B2] Alías-Villegas C. Cubo M. T. Lara-Dampier V. Bellogín R. A. Camacho M. Temprano F. (2015). Rhizobial strains isolated from nodules of *Medicago marina* in southwest Spain are abiotic-stress tolerant and symbiotically diverse. *Syst. Appl. Microbiol.* 38 506–514. 10.1016/j.syapm.2015.07.003 26299372

[B3] Altschul S. F. Gish W. Miller W. Meyers E. W. Lipmann D. J. (1990). Basic local alignment search tool. *J. Mol. Biol.* 215 403–410. 2231712 10.1016/S0022-2836(05)80360-2

[B4] Amgarten D. Martins L. F. Lombardi K. C. Antunes L. P. de Souza A. P. S. Nicastro G. G. (2017). Three novel *Pseudomonas* phages isolated from composting provide insights into the evolution and diversity of tailed phages. *BMC Genom.* 18:346. 10.1186/s12864-017-3729-z 28472930 PMC5418858

[B5] Arndt D. Grant J. Marcu A. Sajed T. Pon A. Liang Y. (2016). PHASTER: a better, faster version of the PHAST phage search tool. *Nucleic Acids Res.* 44 W16–W21. 10.1093/nar/gkw387 27141966 PMC4987931

[B6] Aziz R. K. Ackermann H.-W. Petty N. K. Kropinski A. M. (2018). “Essential steps in characterizing bacteriophages: biology, taxonomy, and genome analysis,” in *Bacteriophages: Methods and Protocols, Vol. III. Methods in Molecular Biology*, Vol. 1681 eds Clokie M. R. J. Kropinski A. M. Lavigne R. (Totowa, NJ: Humana Press), 197–215. 10.1007/978-1-4939-7343-9-15 29134597

[B7] Bailly-Bechet M. Vergassola M. Rocha E. (2007). Causes for the intriguing presence of tRNAs in phages. *Genom. Res.* 17 1486–1495. 10.1101/gr.6649807 17785533 PMC1987346

[B8] Barnet Y. M. (1972). Bacteriophages of *Rhizobium trifolii*. I. morphology and host range. *J. Gen. Virol.* 15 1–15. 10.1099/0022-1317-15-1-1 5025751

[B9] Béna G. Lyet A. Huguet T. Olivieri I. (2005). Medicago – Sinorhizobium symbiotic specificity evolution and the geographic expansion of *Medicago*. *J. Evol. Biol.* 18 1547–1558. 10.1111/j.1420-9101.2005.00952.x 16313467

[B10] Beringer J. E. (1974). R factor transfer in *Rhizobium leguminosarum*. *J. Gen. Microbiol.* 84 188–198. 10.1099/00221287-84-1-188 4612098

[B11] Born Y. Fieseler L. Marazzi J. Lurz R. Duffy B. Loessner M. J. (2011). Novel virulent and broad-host-range *Erwinia amylovora* bacteriophages reveal a high degree of mosaicism and a relationship to *Enterobacteriaceae* phages. *Appl. Environ. Microbiol.* 77 5945–5954. 10.1128/AEM.03022-10 21764969 PMC3165370

[B12] Brewer T. E. Stroupe M. E. Jones K. M. (2014). The genome, proteome and phylogenetic analysis of *Sinorhizobium meliloti* phage (M12, the founder of a new group of T4-superfamily phages. *Virology* 450–451 84–97. 10.1016/j.virol.2013.11.027 24503070

[B13] Brewer T. E. Washburn B. K. Lynn J. S. Jones K. M. (2018). Complete genome sequence of *Sinorhizobium* phage (M6, the first terrestrial phage of a marine phage group. *Microbiol. Resour. Announc.* 7:e001143-18. 10.1128/MRA.01143-18 30533689 PMC6256558

[B14] Bromfield E. E. S. P. Sinha I. B. Wolynetz M. S. (1986). Influence of location, host cultivar, and inoculation on the composition of naturalized populations of *Rhizobium meliloti* in *Medicago sativa* nodules. *Appl. Environ. Microbiol.* 51 1077–1084. 10.1128/aem.51.5.1077-1084.1986 16347054 PMC239014

[B15] Canchaya C. Fournous G. Chibani-Chennoufi S. Dillmann M. L. Brüssow H. (2003). Phage as agents of lateral gene transfer. *Curr. Opin. Microbiol.* 6 417–424. 10.1016/S1369-5274(03)00086-9 12941415

[B16] Carter R. H. Demidenko A. A. Hattingh-Willis S. Rothman-Denes L. B. (2003). Phage N4 RNA polymerase II recruitment to DNA by a single-stranded DNA-binding protein. *Gen. Dev.* 17 2334–2345. 10.1101/gad.1121403 12975320 PMC196469

[B17] Casadesús J. Olivares J. (1979). General transduction in *Rhizobium meliloti* by a thermosensitive mutant of bacteriophage DF2. *J. Bacteriol.* 139 316–317. 10.1128/jb.139.1.316-317.1979 457604 PMC216863

[B18] Casjens S. (2003). Prophages and bacterial genomics: what have we learned so far? *Mol. Microbiol.* 49 277–300. 10.1046/j.1365-2958.2003.03580.x 12886937

[B19] Ceyssens P. J. Brabban A. Rogge L. Lewis M. S. Pickard D. Goulding D. (2010). Molecular and physiological analysis of three *Pseudomonas aeruginosa* phages belonging to the “N4-like viruses”. *Virology* 405 26–30. 10.1016/j.virol.2010.06.011 20619867 PMC3715699

[B20] Chan J. Z. M. Millard A. D. Mann N. H. Schäfer H. (2014). Comparative genomics defines the core genome of the growing N4-like phage genus and identifies N4-like Roseophage specific genes. *Front. Microbiol.* 5:506. 10.3389/fmicb.2014.00506 25346726 PMC4193335

[B21] Choi K. H. McPartland J. Kaganman I. Bowman V. D. Rothman-Denes L. B. Rossman M. G. (2008). Insight into DNA and protein transport in double-stranded DNA viruses: the structure of bacteriophage N4. *J. Mol. Biol.* 378 726–736. 10.1016/j.jmb.2008.02.059 18374942 PMC2396777

[B22] Clokie M. R. J. Millard A. D. Letarov A. V. Heaphy S. (2011). Phages in nature. *Bacteriophage* 1 31–45. 10.4161/bact.1.1.14942 21687533 PMC3109452

[B23] Crockett J. T. Hodson T. S. Hyde J. R. Schouten J. T. Smith T. A. Merill B. D. (2015). *Sinorhizobium* phage phiM9, complete genome, KR052481. *Microbiol. Mol. Biol.*

[B24] Crook M. B. Draper A. L. Guillory R. J. Griffitts J. S. (2013). The *Sinorhizobium meliloti* essential porin RopA1 is a target for numerous bacteriophages. *J. Bacteriol.* 195 3663–3671. 10.1128/JB.00480-13 23749981 PMC3754576

[B25] Cubo M. T. Buendía-Clavería A. M. Beringer J. E. Ruiz-Sainz J. E. (1988). Melanine production by *Rhizobium* strains. *Appl. Environ. Microbiol.* 54 1812–1817. 16347690 10.1128/aem.54.7.1812-1817.1988 PMC202750

[B26] de Maagd R. A. Mulders I. H. Canter Cremers H. C. Lugtenberg B. J. (1992). Cloning, nucleotide sequencing, and expression in *Escherichia coli* of a *Rhizobium leguminosarum* gene encoding a symbiotically repressed outer membrane protein. *J. Bacteriol.* 174 214–221. 10.1128/jb.174.1.214-221.1992 1370281 PMC205698

[B27] Deak V. Lukàcs R. Buzás Z. Pálvölgyi A. Papp P. P. Orosz L. (2010). Identification of tail genes in the temperate phage *16-3* of *Sinorhizobium meliloti* 41. *J. Bacteriol.* 192 1617–1623. 10.1128/JB.01335-09 20081029 PMC2832519

[B28] Decewicz P. Radlinska M. Dziewit L. (2017). Characterization of *Sinorhizobium* sp. LM21 prophages and virus-encoded DNA methyltransferases in the light of comparative genomic analysis of sinorhizobial virome. *Viruses* 9:161. 10.3390/v9070161 28672885 PMC5537653

[B29] Dowdle S. F. Bohlool B. B. (1985). Predominance of fast-growing *Rhizobium japonicum* in a soybean field in the People’s Republic of China. *Appl. Environ. Microbiol.* 50 1171–1176. 10.1128/aem.50.5.1171-1176.1985 16346926 PMC238719

[B30] Dziewit L. Oscik K. Bartosik D. Radlinska M. (2014). Molecular Characterization of a novel temperate *Sinorhizobium* bacteriophage, (LM21, encoding DNA methyltransferase with CcrM-like specificity. *J. Virol.* 88 13111–13124. 10.1128/JVI.01875-14 25187538 PMC4249059

[B31] Fan H. Fan H. An X. Huang Y. Zhang Z. Mi Z. (2012). Complete genome sequence of IME11, a new N4-like bacteriophage. *J. Virol.* 86:13861. 10.1128/JVI.02684-12 23166261 PMC3503130

[B32] Fan L. M. Ma Z. Q. Liang J. Q. Li H. F. Wang E. T. Wei G. H. (2011). Characterization of a copper-resistant symbiotic bacterium isolated from *Medicago lupine* growing in mine tailings. *Bioresour. Technol.* 102 703–709. 10.1016/j.biortech.2010.08.046 20843682

[B33] Finan T. M. Hartweig E. LeMieux K. Bergman K. Walker G. C. Signer E. R. (1984). General transduction in *Rhizobium meliloti*. *J.Bacteriol.* 159 120–124. 10.1128/jb.159.1.120-124.1984 6330024 PMC215601

[B34] Forrai T. Vincze E. Banfalvi Z. Kiss G. B. Randhawa G. S. Kondorosi A. (1983). Localization of symbiotic mutations in *Rhizobium meliloti*. *J. Bacteriol.* 153 635–643. 10.1128/jb.153.2.635-643.1983 6296048 PMC221679

[B35] Ganyu A. Csiszovszki Z. Ponyi T. Kern A. Buzás Z. Orosz L. (2005). Identification of cohesive ends and genes encoding the terminase of phage 16-3. *J. Bacteriol.* 187 2526–2531. 10.1128/JB.187.7.2526-2631.2005 15774897 PMC1065242

[B36] Gibson K. E. Kobayashi H. Walker G. C. (2008). Molecular determinants of a symbiotic chronic infection. *Annu. Rev. Genet.* 42 413–441. 10.1146/annurev.genet.42.110807.091427 18983260 PMC2770587

[B37] Hendrix R. W. (2002). Bacteriophages: evolution of the majority. *Theor. Popul. Biol.* 61 471–480. 10.1006/tpbi.2002.1590 12167366

[B38] Hendrix R. W. Smith M. C. M. Burns R. N. Ford M. E. Hatfull G. F. (1999). Evolutionary relationships among diverse bacteriophages and prophages: all the world’s a phage. *Proc. Natl. Acad. Sci. U.S.A.* 96 2192–2197. 10.1073/pnas.96.5.2192 10051617 PMC26759

[B39] Hodson T. S. Hyde J. R. Schouten J. T. Crockett J. T. Smith T. A. Merrill B. D. (2015). *Sinorhizobium* phage phiN3, complete genome, KR052482. *Microbiol. Mol. Biol.*

[B40] Hyman P. Abedon S. T. (2009). “Practical methods for determining phage growth parameters,” in *Bacteriophages, Methods and Protocols. Vol. I: Isolation, Characterization, and Interactions*, eds Clokie M. R. J. Kropinski A. M., (Totowa, NJ: Humana Press), 175–202. 10.1007/978-1-60327-164-6 19066822

[B41] Hyman P. Abedon S. T. (2010). Bacteriophage host range and bacterial resistance. *Adv. Appl. Microbiol.* 70 217–248. 10.1016/S0065-2164(10)70007-1 20359459

[B42] Johnson M. C. Sena-Velez M. Washburn B. K. Platt G. N. Lu S. Brewer T. E. (2017). Structure, proteome and genome of *Sinorhizobium meliloti* phage (M5: a virus with LUZ24-like morphology and a highly mosaic genome. *J. Struct. Biol.* 200 343–359. 10.1016/j.jsb.2017.08.005 28842338

[B43] Johnson M. C. Tatum K. B. Lynn J. S. Brewer T. E. Lu S. Washburn B. K. (2015). *Sinorhizobium meliloti* phage (M9 defines a new group of T4 superfamily phages with unusual genomic features but a common T=16 capsid. *J. Virol.* 89 10945–10958. 10.1128/JVI.01353-15 26311868 PMC4621102

[B44] Jun J. W. Yun S. K. Kim H. J. Chai J. Y. Park S. C. (2014). Characterization and complete genome sequence of a novel N4-like bacteriophage, pSb-1 infecting *Shigella boydii*. *Res. Microbiol.* 165 671–678. 10.1016/j.resmic.2014.09.006 25283727

[B45] Kankila J. Lindström K. (1994). Host range, morphology and DNA restriction patterns of bacteriophage isolates infecting *Rhizobium leguminosarum* bv. *trifolii*. *Soil Biol. Biochem.* 26 429–437. 10.1016/0038-0717(94)90174-0

[B46] Kowalski M. (1967). Transduction in *Rhizobium meliloti*. *Acta Microbiol. Pol.* 16 7–12. 4166074

[B47] Kropinski A. M. (2009). Measurement of the rate of attachment of bacteriophage to cells. *Methods Mol Biol.* 501 151–156. 10.1007/978-1-60327-164-6 19066819

[B48] Kropinski A. M. (2018). Practical advice on the One-step growth curve. *Methods Mol. Biol.* 1681 41–47. 10.1007/978-1-4939-7343-9_3 29134585

[B49] Kropinski A. M. Mazzocco A. Waddell T. E. Lingohr E. Johnson R. P. (2009). Enumeration of bacteriophages by double agar overlay plaque assay. *Methods Mol Biol.* 501 69–76. 10.1007/978-1-60327-164-6 19066811

[B50] Krsmanovic-Simic D. Werquin M. (1973). Étude des bacteriophages de *Rhizobium meliloti*. *C.R. Hebd. Seances Acad. Sci. Ser. D Sci. Natur.* 276 2745–2748. 4198859

[B51] Kwiatek M. Parasion S. Rutyna P. Mizak L. Gryko R. Niemcewicz M. (2017). Isolation of bacteriophages and their application to control *Pseudomonas aeruginosa* in planktonic and biofilm models. *Res. Microbiol.* 168 194–207. 10.1016/j.resmic.2016.10.009 27818282

[B52] Lavigne R. Seto D. Mahadevan P. Ackermann H.-W. Kropinski M. (2008). Unifying classical and molecular taxonomic classification: analysis of the *Podoviridae* using BLASTP-based tools. *Res. Microbiol.* 159 406–414. 10.1016/j.resmic.2008.03.005 18555669

[B53] Lenneman B. R. Rothman-Denes L. B. (2015). Structural and biochemical investigation of bacteriophage N4-encoded RNA polymerases. *Biomolecules* 5 647–667. 10.3390/biom5020647 25924224 PMC4496689

[B54] Lowe T. M. Chan P. P. (2016). tRNAscan-SE On-line: integrating search and context for analysis of transfer RNA genes. *Nucl. Acids Res.* 44 W54–W57. 10.1093/nar/gkw413 27174935 PMC4987944

[B55] Malek W. Wdowiak-Wróbel S. Bartosik M. Konopa G. Narajczyk M. (2009). Characterization of phages virulent for *Robinia pseudoacacia* rhizobia. *Curr Microbiol.* 59 187–192. 10.1007/s00284-009-9421-z 19459003

[B56] Martin M. O. Long S. R. (1984). Generalized transduction in *Rhizobium meliloti*. *J. Bacteriol.* 159 125–129. 10.1128/jb.159.1.125-129.1984 6330025 PMC215602

[B57] Martínez E. Pardo M. Palacios R. Cevallos M. (1985). Reiteration of nitrogen fixation gene sequences and specificity of *Rhizobium* in nodulation and nitrogen fixation in *Phaseolus vulgaris*. *J. Gen. Microbiol.* 131 1779–1786. 10.1099/00221287-131-7-1779

[B58] Meade H. M. Long S. R. Ruvkun G. B. Brown S. E. Ausubel F. M. (1982). Physical and genetic characterization of symbiotic and auxotrophic mutants of *Rhizobium meliloti* induced by transposon Tn*5* mutagenesis. *J. Bacteriol.* 149 114–122. 10.1128/jb.149.1.114-122.1982 6274841 PMC216598

[B59] Meade H. M. Signer E. R. (1977). Genetic mapping of *Rhizobium meliloti*. *Proc. Natl. Acad. Sci. U.S.A.* 74 2076–2078. 10.1073/pnas.74.5.2076 266730 PMC431077

[B60] Mendum T. A. Clark I. M. Hirsch P. R. (2001). Characterization of two novel *Rhizobium leguminosarum* bacteriophages from a field release site of genetically-modified rhizobia. *Ann. Leeuw.* 79 189–197. 11520005 10.1023/a:1010238412538

[B61] Moreno Switt A. I. Orsi R. H. den Bakker H. C. Vongkamjan K. Altier C. Wiedmann M. (2013). Genomic characterization provides new insight into *Salmonella* phage diversity. *BMC Genom.* 14:481. 10.1186/1471-2164-14-481 23865498 PMC3728262

[B62] Morris P. Marinelli L. J. Jacobs-Sera D. Hendrix R. W. Hatfull G. F. (2008). Genomic characterization of mycobacteriophage giles: evidence for phage acquisition of host DNA by illegitimate recombination. *J. Bacteriol.* 190 2172–2182. 10.1128/JB.01657-07 18178732 PMC2258872

[B63] Paddison P. Abedon S. T. Dressman H. K. Gailbreath K. Tracy J. Mosser E. (1998). The roles of the bacteriophage T4 *r* genes in lysis inhibition and fine-structure genetics: a new perspective. *Genetics* 148 1539–1550. 9560373 10.1093/genetics/148.4.1539 PMC1460109

[B64] Park J. Lee G. M. Kim D. Park D. H. Oh C.-S. (2018). Characterization of the lytic bacteriophage phiEaP-8 effective against both *Erwinia amylovora* and *Erwinia pyrifoliae* causing severe diseases in apple and pear. *Plant Pathol. J.* 34 445–450. 10.5423/PPJ.NT.06.2018.0100 30369854 PMC6200048

[B65] Pedulla M. L. Ford M. E. Houtz J. M. Karthikeyan T. Wadsworth C. Lewis J. A. (2003). Origins of highly mosaic mycobacteriophage genomes. *Cell* 173 171–182. 10.1016/s0092-8674(03)00233-2 12705866

[B66] Peng Q. Yuan Y. (2018). Characterization of a newly isolated phage infecting pathogenic *Escherichia coli* and analysis of its mosaic structural genes. *Sci. Rep.* 8:8086. 10.1038/s41598-018-26004-4 29795390 PMC5967307

[B67] Pérez-Montaño F. Alías-Villegas C. Bellogín R. A. del Cerro P. Espuny M. R. Jiménez-Guerrero I. (2014). Plant growth promotion in cereal and leguminous agricultural important plants: from microorganism capacities to crop production. *Microbiol. Res.* 169 325–336. 10.1016/j.micres.2013.09.011 24144612

[B68] Petty N. K. Foulds I. J. Pradel E. Ewbank J. J. Salmond G. P. C. (2006). A generalized transducing phage (>(IF3) for the genomically sequenced *Serratia marcescens* strain Db11: a tool for functional genomics of an opportunistic human pathogen. *Microbiology* 152 1701–1708. 10.1099/mic.028712-0 16735733

[B69] Rakhuba D. V. Kolomiets E. I. Szwajcer Dey E. Novik G. I. (2010). Bacteriophage receptors, mechanisms of phage adsorption and penetration into host cell. *Pol. J. Microbiol.* 59 145–155. 10.33073/pjm-2010-023 21033576

[B70] Rodríguez-Navarro D. N. Bellogín R. Camacho M. Daza A. Medina C. Ollero F. J. (2003). Field assessment and genetic stability of *Sinorhizobium fredii* strain SMH12 for commercial soybean inoculants. *Eur. J. Agron.* 19 299–309. 10.1016/s1161-0301(02)00076-x

[B71] Rome S. Fernández M. P. Brunel B. Normand P. Cleyet-Marel J.-C. (1996). *Sinorhizobium medicae* sp. Nov., isolated from annual *Medicago* spp. *Int. J. Syst. Bacteriol.* 46 972–980. 10.1099/00207713-46-4-972 8863426

[B72] Saitou N. Nei M. (1987). The neighbor-joining method: a new method for reconstructing phylogenetic trees. *Mol. Biol. Evol.* 4 406–425. 10.1093/oxfordjournals.molbev.a040454 3447015

[B73] Sambrook J. Russell D. W. (2001). *Molecular Cloning: A Laboratory Manual*, 3rd Edn, Cold Spring Harbor Laboratory Press.

[B74] Santamaría R. I. Bustos P. Sepúlveda-Robles O. Lozano L. Rodríguez C. Fernández J. L. (2014). Narrow-host-range bacteriophages that infect *Rhizobium etli* associate with distinct genomic types. *Appl. Environ. Microbiol.* 80 446–454. 10.1128/AEM.02256-13 24185856 PMC3911081

[B75] Schouten J. T. Crockett J. T. Hodson T. S. Hyde J. R. Smith T. A. Merrill B. D. (2015). *Sinorhizobium Phage phiM3, Complete Genome, KR052480. Microbiology Molecular. Biology.* Provo, UT: Brigham Young University.

[B76] Schulmeister S. A. Krol J. E. Vorhoelter F. J. Skorupska A. M. Lotz W. (2009). Sequence of the genome of *Sinorhizobium meliloti* bacteriophage PBC5, NC_003324.1. *Nat. Center Biotechnol. Inform.*

[B77] Sharma R. S. Mohmmed A. Babu C. R. (2002). Diversity among rhizobiophages from rhizospheres of legumes inhabiting three ecogeographical regions of India. *Soil Biol. Biochem.* 34 965–973. 10.1016/s0038-0717(02)00030-5

[B78] Sik T. Harwath J. Chatterjee S. (1980). Generalized transduction in *Rhizobium meliloti*. *Mol. Gen. Genet.* 178 511–516. 10.1007/bf00337855 6930536

[B79] Stroupe M. E. Brewer T. E. Sousa D. R. Jones K. M. (2014). The structure of *Sinorhizobium meliloti* phage (M12, which has a novel *T* = 19l triangulation number and is the founder of a new group of T4-superfamily phages. *Virology* 450-451 205–212. 10.1016/j.virol.2013.11.019 24503083

[B80] Swinton D. Hattman S. Benzinger R. Buchanan-Wollaston V. Beringer J. (1985). Replacement of the deoxycytidine residues in *Rhizobium* bacteriophage RL38JI DNA. *FEMS Lett.* 184 294–298. 10.1016/0014-5793(85)80625-6 2987032

[B81] Tamura K. Peterson D. Peterson N. Stecher G. Nei M. Kumar S. (2011). MEGA5: molecular evolutionary genetics analysis using maximum likelihood, evolutionary distance, and maximum parsimony methods. *Mol. Biol. Evol.* 28 2731–2739. 10.1093/molbev/msr121 21546353 PMC3203626

[B82] Trinick M. J. (1980). Relationship among the fast-growing rhizobia of *Lablab purpureus*, *Leucaena leucocephala*, *Mimosa* spp., *Acacia farnesiana* and *Sesbania grandiflora* and their affinities with other rhizobial group. *J. Appl. Bacteriol.* 49 39–53. 10.1111/j.1365-2672.1980.tb01042.x

[B83] Vandecaveye S. C. Katznelson H. (1936). Bacteriophage as related to the root nodule bacteria of alfalfa. *J. Bacteriol.* 31 465–477. 10.1128/jb.31.5.465-477.1936 16559902 PMC543734

[B84] Werquin M. Ackermann H.-W. Levesque R. C. (1988). A study of 33 bacteriophages of *Rhizobium meliloti*. *Appl. Environ. Microbiol.* 54 188–196. 10.1128/aem.54.1.188-196.1988 16347525 PMC202420

[B85] Willems A. (2006). The taxonomy of rhizobia: an overview. *Plant Soil* 287 3–14. 10.1007/s11104-006-9058-7 7708010

[B86] Willis S. H. Kazmierczak K. M. Carter R. H. Rothman-Denes L. B. (2002). N4 RNA polymerase II, a heterodimeric RNA polymerase with homology to the single-subunit family of RNA polymerases. *J. Bacteriol.* 184 4952–4961. 10.1128/JB.184.18.4952-4961.2002 12193610 PMC135322

[B87] Wittmann J. Dreiseikelmann B. Rohde M. Meier-Kolthoff J. P. Bunk B. Rohde C. (2014). First genome sequences of *Achromobacter* phages reveal new members of the N4 family. *Virol. J.* 11:14. 10.1186/1743-422X-11-14 24468270 PMC3915230

[B88] Wittmann J. Klumpp J. Moreno Switt A. I. Yagubi A. Ackermann H.-W. Wiedmann M. (2015). Taxonomic reassessment of N4-like viruses using comparative genomics and proteomics suggests a new subfamily – “*Enquartavirinae*”. *Arch. Virol.* 160 3053–3062. 10.1007/s00705-015-2609-6 26395091

[B89] Young J. M. (2003). The genus name *Ensifer* Casida 1982 takes priority over *Sinorhizobium* Chen et al. 1988, and *Sinorhizobium morelense* Wang et al. 2002 is a later synonym of *Ensifer adhaerens* Casida 1982. Is the combination “*Sinorhizobium adherens*” (Casida 1982) Willems et al. 2003 legitime? Request for an Opinion. *Int. J. Syst. Evol. Microbiol.* 53 2107–2110. 10.1099/ijs.0.02665-0 14657154

[B90] Zhang Y. M. Tian C. F. Sui X. H. Chen W. F. Chen W. X. (2012). Robust markers reflecting phylogeny and taxonomy of rhizobia. *PLoS One* 7:e44936. 10.1371/journal.pone.0044936 23028691 PMC3444505

[B91] Zhao Y. Wang K. Jiao N. Chen F. (2009). Genome sequences of two novel phages infecting marine roseobacters. *Environ. Microbiol.* 11 2055–2064. 10.1111/j.1462-2920.2009.01927.x 19689706 PMC2784036

