# Supplementary material for: Diversity of Sinorhizobium (Ensifer) meliloti Bacteriophages in the Rhizosphere of Medicago marina: Myoviruses, Filamentous and N4-Like Podovirus
Source: Front Microbiol. 2020 Jan 24;11:22. doi: 10.3389/fmicb.2020.00022 (PMC6992544; doi:10.3389/fmicb.2020.00022)
Supplement: Supplementary file 1 [file Image_1.pdf]

Fig. S1A

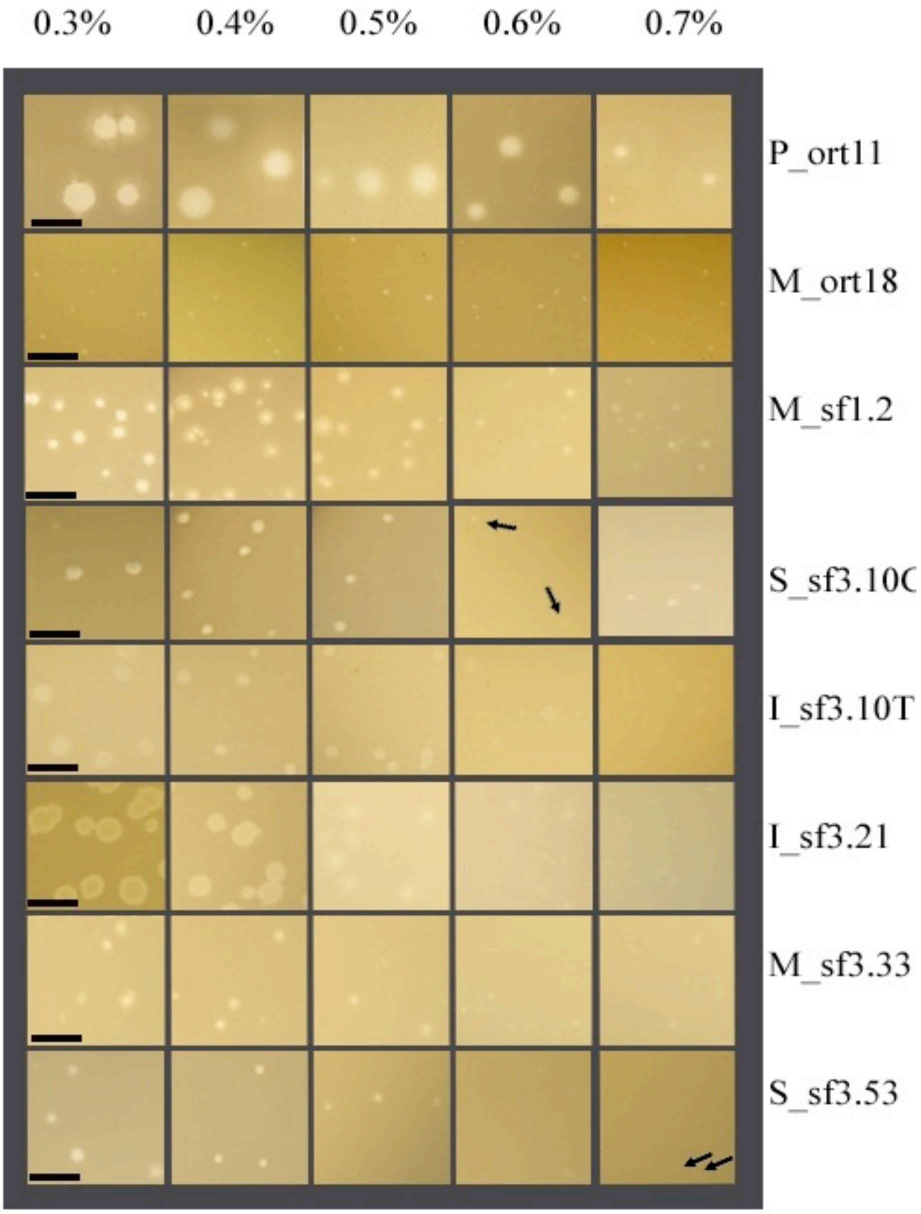

Fig. S1B

	**Size (mm) of plaques on increasing percentage (wt/v) of top TY-agar**				
**Phage**	**0.3%**	**0.4%**	**0.5%**	**0.6%**	**0.7%**
P_ort11	2.45±0.6	2.2±0.4	0.35±0.9	1.3±0.4	1.05±0.07
M_ort18	0.55±0.3	0.55±0.3	0.45±0.2	0.35±0.2	0.3±0.2
M_sf1.2	1.05±0.2	0.8±0.5	0.75±0.5	0.6±0.4	0.45±0.4
S_sf3.10C	1.95±0.07	1.4±0.1	1.05±0.07	1.15±0.07	0.8±0.2
I_sf3.10T	1.6±0.4	0.9±0.1	0.95±0.2	0.95±0.2	0.7±0.2
I_sf3.21	1.75±0.3	1.6±0.5	1.25±0.3	0.9±0.1	0.6±0.01
M_sf3.33	0.8±0.5	0.65±0.5	0.6±0.2	0.15±0.07	0.1±0.01
S_sf3.53	1±0.1	0.8±0.1	0.7±0.2	0.3±0.1	0.1±0.01

Supplementary Figure S1. Phage-plaques sizes on increasing percentages of TY-agar (%, w/v) in the top lawn overlays. Bars, 4 mm. (**A**) photographs of plaques (**B**) sizes of them in mm. Arrows indicate very small plaques.
