# Supplementary material for: Diversity of Sinorhizobium (Ensifer) meliloti Bacteriophages in the Rhizosphere of Medicago marina: Myoviruses, Filamentous and N4-Like Podovirus
Source: Front Microbiol. 2020 Jan 24;11:22. doi: 10.3389/fmicb.2020.00022 (PMC6992544; doi:10.3389/fmicb.2020.00022)
Supplement: Supplementary file 2 [file Image_2.pdf]

Fig. S2

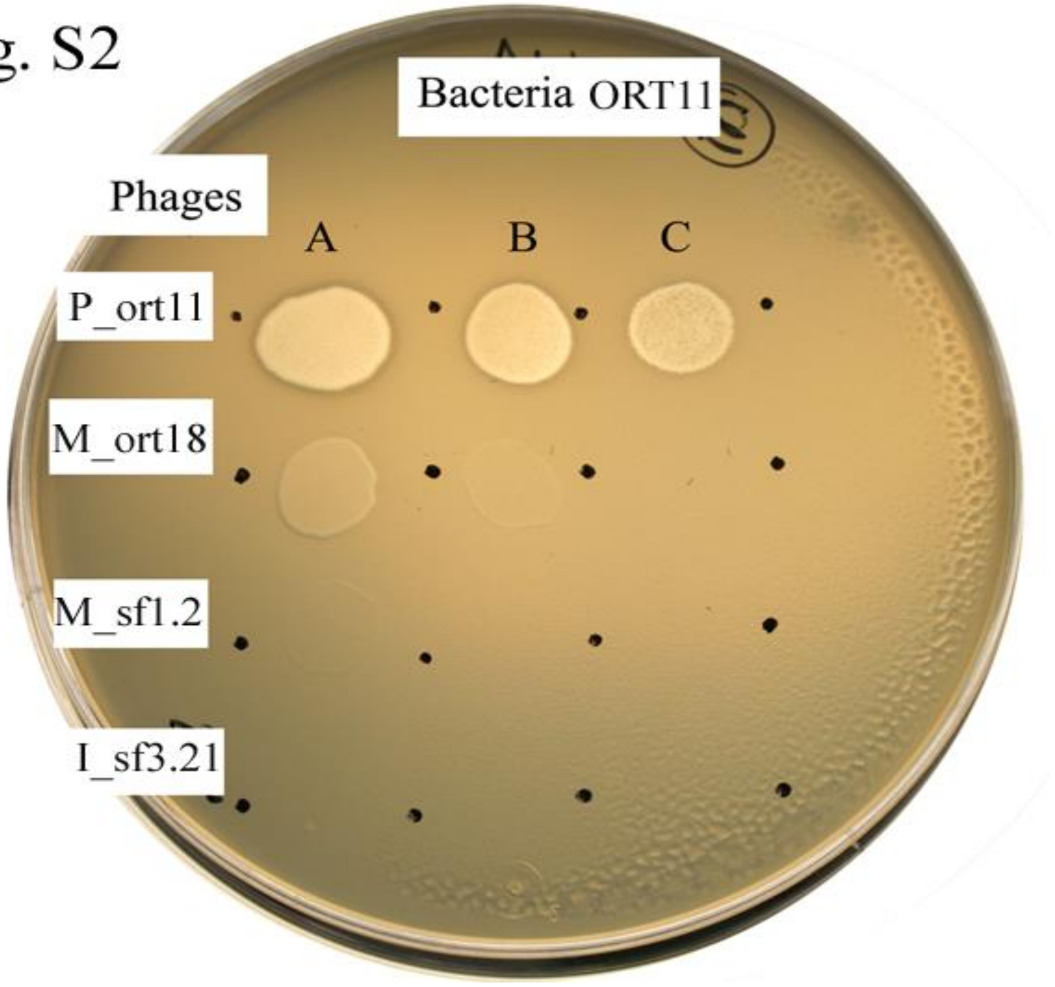

Supplementary Figure S2. **Host range of bacteria *Sinorhizobium meliloti* ORT11 to phages P_ort11, M_ort18, _M_sf1.2, and I_sf3.21**. Bacterial lawns were propagated by adding 200 μl of overnight culture to the 0.4% (wt/v) top-TY agar and pouring onto a solid TY plate. After 15 min, 10 μl of droplets of lysates were added without dilute (A), 10 times diluted (B), and 100 times diluted (C). Plates were incubated 2 days at 28ºC for the presence of lysis plaques
