# Supplementary material for: Diversity of Sinorhizobium (Ensifer) meliloti Bacteriophages in the Rhizosphere of Medicago marina: Myoviruses, Filamentous and N4-Like Podovirus
Source: Front Microbiol. 2020 Jan 24;11:22. doi: 10.3389/fmicb.2020.00022 (PMC6992544; doi:10.3389/fmicb.2020.00022)
Supplement: Supplementary file 3 [file Image_3.pdf]

Fig. S3A

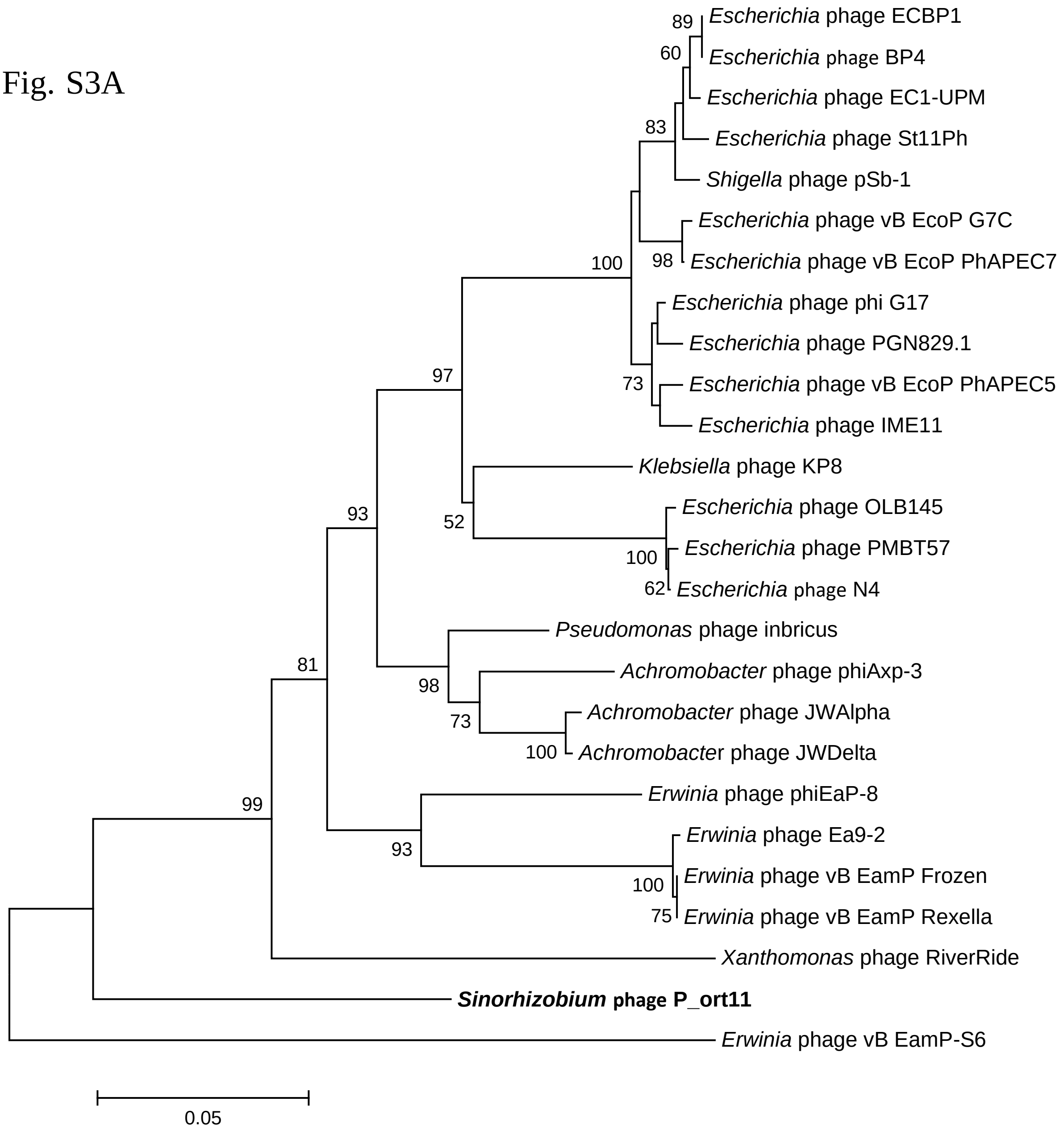
Escherichia phage ECBP1
Escherichia phage BP4
Escherichia phage EC1-UPM
Escherichia phage St11Ph
Shigella phage pSb-1
Escherichia phage vB EcoP G7C
Escherichia phage vB EcoP PhAPEC7
Escherichia phage phi G17
Escherichia phage PGN829.1
Escherichia phage vB EcoP PhAPEC5
Escherichia phage IME11
Klebsiella phage KP8
Escherichia phage OLB145
Escherichia phage PMBT57
Escherichia phage N4
Pseudomonas phage inbricus
Achromobacter phage phiAxp-3
Achromobacter phage JWAlpha
Achromobacter phage JWDelta
Erwinia phage phiEaP-8
Erwinia phage Ea9-2
Erwinia phage vB EamP Frozen
Erwinia phage vB EamP Rexella
Xanthomonas phage RiverRide
Sinorhizobium phage P_ort11
Erwinia phage vB EamP-S6
89
60
83
100
98
73
97
93
52
100
62
81
98
73
100
99
93
100
75
0.05

Fig. S3B

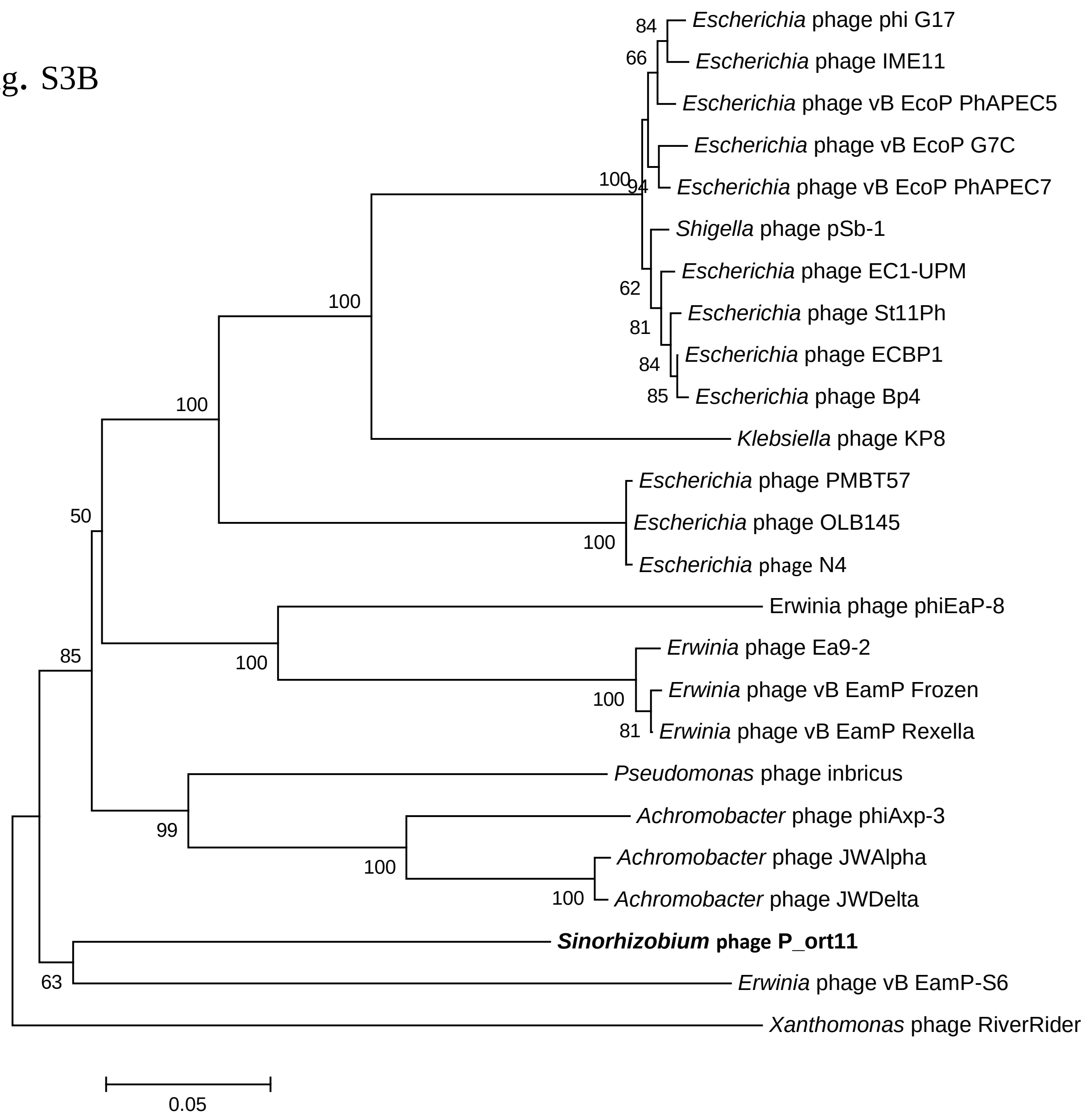
Escherichia phage phi G17
Escherichia phage IME11
Escherichia phage vB EcoP PhAPEC5
Escherichia phage vB EcoP G7C
Escherichia phage vB EcoP PhAPEC7
Shigella phage pSb-1
Escherichia phage EC1-UPM
Escherichia phage St11Ph
Escherichia phage ECBP1
Escherichia phage Bp4
Klebsiella phage KP8
Escherichia phage PMBT57
Escherichia phage OLB145
Escherichia phage N4
Erwinia phage phiEaP-8
Erwinia phage Ea9-2
Erwinia phage vB EamP Frozen
Erwinia phage vB EamP Rexella
Pseudomonas phage inbricus
Achromobacter phage phiAxp-3
Achromobacter phage JWAlpha
Achromobacter phage JWDelta
Sinorhizobium phage P_ort11
Erwinia phage vB EamP-S6
Xanthomonas phage RiverRider
84
66
100
94
62
81
84
85
100
100
50
100
100
100
85
99
100
100
63
0.05

Fig. S3C

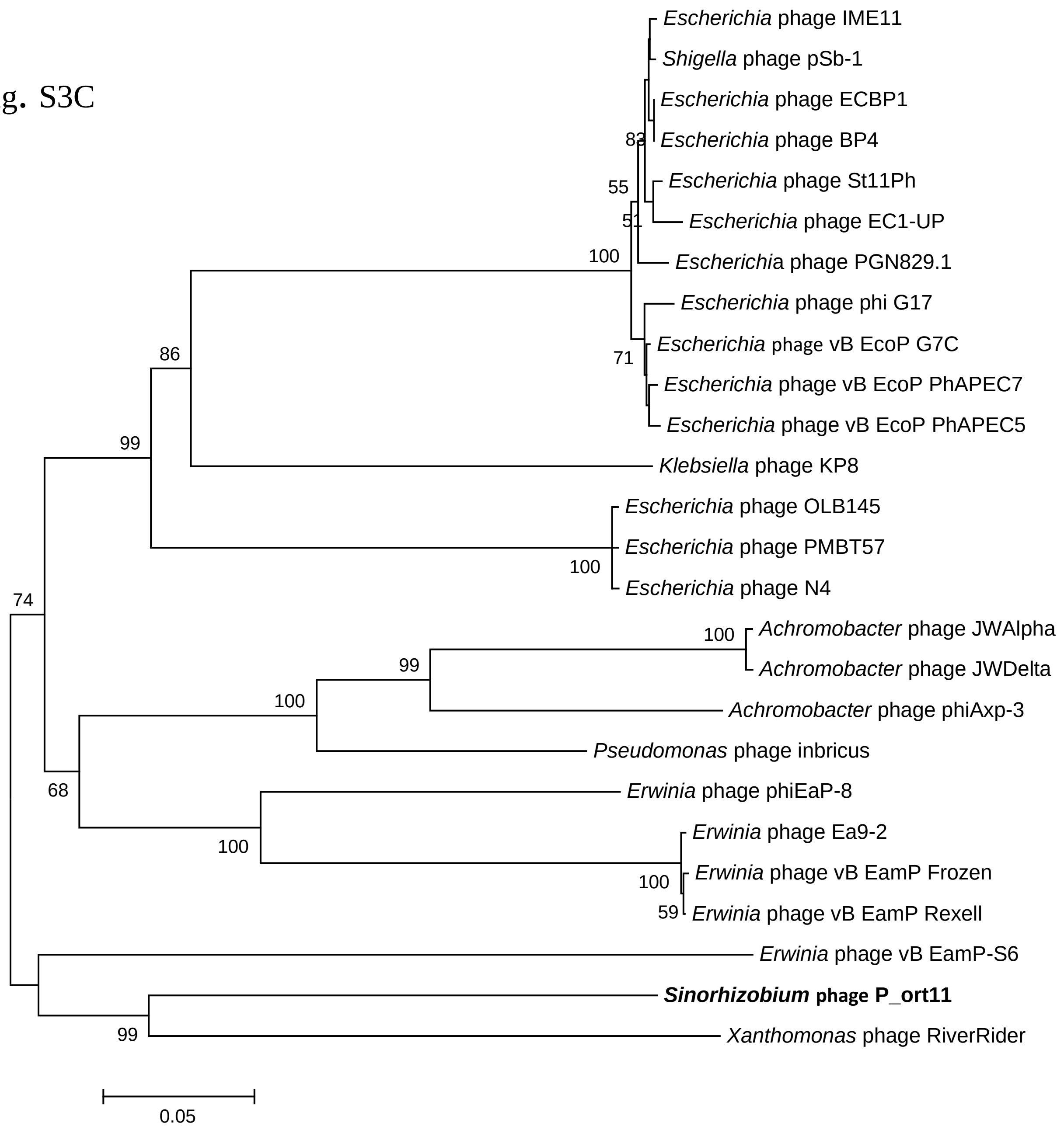

Supplementary Figure S3. Phylogenetic trees of the major capsid protein (S3A), portal protein (S3B), and DNA polymerase (S3C) of P_ort11 compared to other N4-like phages. Neigbour-joining tree was constructed based on ClustalW (MEGA5). Bootstrap values (>50%) are expressed as percentages of 1,000 replicated. Bar, 0.05 amino acid substitutions per site.
