# Supplementary material for: Diversity of Sinorhizobium (Ensifer) meliloti Bacteriophages in the Rhizosphere of Medicago marina: Myoviruses, Filamentous and N4-Like Podovirus
Source: Front Microbiol. 2020 Jan 24;11:22. doi: 10.3389/fmicb.2020.00022 (PMC6992544; doi:10.3389/fmicb.2020.00022)
Supplement: Supplementary file 4 [file Table_1.pdf]

Supplementary Table S1. **Sensitivity of P_ort11 virions to temperature, pH and NaCl concentration**

		% of viability of virions
**4ºC**	**pH 7.5**	100±0
	**pH 8.5**	91.07±5.68
	**pH 5.5**	79.17±18.43
	**300 mM**	89.85±1.33
	**600 mM**	81.59±8.91
	**800 mM**	90.87±13.62
**28ºC**	**pH 7.5**	73.14±6.61
	**pH 8.5**	3.11±2.56
	**pH 5.5**	1.68±2.19
	**300 mM**	9.85±5.97
	**600 mM**	7.39±0.76
	**800 mM**	17.59±3.19
**37ºC**	**pH 7.5**	8.62±8.69
	**pH 8.5**	0.0001±0.002
	**pH 5.5**	0±0
	**300 mM**	0.00095±0.00038
	**600 mM**	0.00085±0.00008
	**800 mM**	0.0059±0.00056
